# Valorization of orange peel by-products via ultrasound-pretreated enzymatic extraction of soluble dietary fiber: Structural and functional characterization

**DOI:** 10.1371/journal.pone.0357376

**Published:** 2026-09-01

**Authors:** Jhonatan D. Quiñonez-Ensuncho, Gala Rosales, Laura Ortega-Ruiz, Alfonso Benítez-Páez, Beatriz E. Valdés-Duque, Álvaro S. Lima, J. Felipe Osorio-Tobón

**Affiliations:** 1 Faculty of Health Sciences, University Institution Colegio Mayor de Antioquia, Medellín, Antioquia, Colombia; 2 Programa de Pós-Graduação em Engenharia Química, Universidade Federal da Bahia, Salvador, Brazil; 3 Bioinformatics, Computational Biology and Data Science Research Unit, Institute of Agrochemistry and Food Technology (IATA-CSIC), Paterna-Valencia, Spain; Prince of Songkla University, THAILAND

## Abstract

Agro-industrial by-products valorization has attracted increasing attention due to their potential as sustainable sources of bioactive compounds, particularly dietary fibers. In this study, orange peel by-products were used as a raw material for the recovery of soluble dietary fiber via enzymatic extraction and ultrasound-pretreated enzymatic extraction. Ultrasound-pretreated enzymatic extraction achieved a soluble dietary fiber recovery of 26.04 ± 0.81% with approximately 90% purity. The content of phenolic compounds bound to soluble dietary fiber ranged from 3.23 ± 0.03 to 12.15 ± 0.21 mg GAE g^-1^. Soluble dietary fiber obtained by alkaline extraction exhibited higher water-holding capacity (9.16 ± 0.08 g g ^-1^). Enzymatic extraction caused structural modifications and partial depolymerization, reducing fiber porosity and water affinity. Oil-holding capacity ranged from 1.08 ± 0.12 to 1.24 ± 0.05 g g^-1^, with no differences among extraction methods. Ultrasound-pretreated enzymatic extraction produced soluble dietary fiber with a more amorphous structure, lower crystallinity, greater surface disruption, and higher thermal stability. Moreover, it is a suitable substrate for the growth of mammalian intestinal symbionts such as *Faecalibaculum rodentium* and *Bacteroides thetaiotaomicron*, species recognized as beneficial for host health. Ultrasound pretreatment is a promising strategy for recovering high-value soluble dietary fibers from orange peel by-products, thereby enabling the production of tailored functional ingredients for food applications.

## Introduction

Orange is one of the most important crops worldwide, with an estimated annual production of 47 million tons in 2024 [1]. Globally, the management of citrus residues is a critical environmental challenge, as millions of tons of organic waste are generated each year, releasing significant amounts of greenhouse gases, including carbon dioxide and methane, during decomposition [2]. These by-products, which include peel, albedo, and endocarps, account for up to 55% of the fruit’s weight. In Colombia, where production reached 756 thousand tons in 2023, the common practice of discarding these residues in landfills represents a substantial loss of biomass rich in carotenoids, flavonoids, pectin, and dietary fiber (DF) [3]. Consequently, in accordance with the principles of the circular economy, it is imperative to develop valorization strategies that mitigate environmental impacts while recovering high-value functional ingredients [4]. DF is a carbohydrate found in plant foods that is not digestible in the human small intestine and is used as an energy source by the microbiota in the large intestine. DF is considered a functional ingredient due to its beneficial properties. For example, DF is recognized for reducing the risk of cardiovascular and diabetes diseases, as well as its positive effects on the intestine, promoting bowel movement and relieving constipation [5]. Based on water solubility, DF is classified as soluble dietary fiber (SDF) and insoluble dietary fiber (IDF). SDF increases viscosity, decelerates gastric emptying, and reduces blood glucose and cholesterol levels. Moreover, SDF stabilizes insulin, improves macronutrient absorption, and reduces the risk of diseases such as cancer and obesity, thereby benefiting metabolism and modulating the microbiota [6]. IDF presents a slow and incomplete fermentation process, which is directly related to intestinal peristalsis, decreased gastrointestinal transit time, and increased fecal volume, facilitating defecation and alleviating constipation [7].

DF can be extracted using biological, chemical, and physical techniques, or their combinations (e.g., physical and biological). Traditional chemical extraction methods (acidic or alkaline) are common due to low cost but often require high temperatures and pose environmental challenges [8]. In contrast, ultrasound-assisted extraction (UAE) is a sustainable “green” technology that operates at lower temperatures with reduced solvent requirements [9]. The core mechanism is acoustic cavitation: sound waves (typically> 20 kHz) create microbubbles that grow and then collapse, releasing high-intensity energy. This generates hydrodynamic shear forces that induce cell wall disruption. This structural modification increases the surface area and improves mass transfer, allowing solvents and reagents to penetrate the internal structure more efficiently, thereby increasing the recovery of DF fractions [10].

On the other hand, the enzymatic extraction (EE) method is considered an effective approach for removing impurities from DF, such as proteins and starch, through enzymatic hydrolysis. This process generally requires protease, α-amylase, and amyloglucosidase. This approach to extraction is suitable for obtaining high-quality dietary fiber, as enzymatic action modifies fiber structure and redistributes its composition [11]. The primary advantages of this method include high extraction efficiency, mild processing conditions, and reduced environmental impact due to minimal hazardous by-product formation [7]. The integration of EE and ultrasound enables the production of fibers with interesting properties at enhanced yields [12].

Conventional techniques (such as acid, alkaline, and hot-water extraction) are often limited by low extraction yields and the risk of polysaccharide degradation from harsh reagents and high temperatures. For example, Bakr et al. [13] reported a modest SDF yield of 5.61% from quince using alkaline extraction. In contrast, green technologies such as ultrasound-assisted enzymatic extraction have demonstrated higher efficiency. Panwar et al. [12] achieved an SDF yield of 12.19% from sweet lime pomace using this approach. These alternative methods represent a significant innovation in fiber purity and preserving structural integrity while adhering to environmentally sustainable practices. Despite these advances, limiting solid-liquid ratios is often employed, restricting the scalability of these technologies for industrial DF recovery. Another critical bottleneck is temperature control during ultrasound-assisted enzymatic extraction. Because the enzymatic process is carried out at temperatures higher than those recommended by the ultrasound equipment supplier, the simultaneous application of ultrasound and enzymatic hydrolysis is limited. Thus, the application of ultrasound as a pretreatment is a valuable alternative. Although previous studies have reported that extraction conditions significantly influence DF quality and properties, there remains a gap in research on the application of ultrasound-pretreated enzymatic extraction of SDF from orange peel by-products (OPB). Moreover, evidence on the application of ultrasound as a pretreatment for SDF recovery from orange residues at low mass-to-volume ratios, such as 1:25, and under temperature-controlled sonication conditions remains limited. This mass-to-volume ratio offers a critical advantage by reducing solvent consumption and minimizing waste generation, thereby facilitating process scalability and industrial implementation. Beyond these operational advantages, the innovative contribution of this work lies in transcending the conventional physicochemical characterization by validating the biological functionality of the recovered SDF. This study aims to evaluate the effects of different extraction methods (alkaline extraction, alkaline extraction with ultrasonic pretreatment, enzymatic extraction, and enzymatic extraction with ultrasonic pretreatment) on the extraction yield of dietary fiber fractions from OPB. The influence of these treatments on the techno-functional and structural properties of SFD was also assessed. Additionally, its potential as a carbon source for the growth of *Faecalibaculum rodentium* ABL288 was evaluated.

## Materials and methods

### Materials

OPB were kindly provided by Fruti Paisa, Medellín, Colombia. The pre-treatment was carried out as described by Ye et al. [14] with some modifications. Briefly, the OPB were first washed with distilled water to remove inorganic residues. They were then submerged in water at 70 °C for 20 min to eliminate low-molecular-weight sugars, organic acids, and inactive enzymes. The OPB were dried in an oven at 50 °C for 48 hours (Memmert, model UN110, Schwabach, Germany). After drying, the peels were milled and sieved using a 50-mesh sieve (0,297 mm). Processed OPB were stored in hermetically sealed bags (Alico S.A., Flex Up, Medellín, Colombia) and protected from light to prevent degradation. Samples were stored at room temperature until fiber extraction. Thermostable α-amylase (12000 U g^-1^) and amyloglucosidase (104000 U g^-1^) were purchased from Proenzymas S.A.S. (Cali, Colombia), and protease (2.4 U g^-1^) was purchased from Sigma-Aldrich. All reagents used in this study were of analytical grade.

### Extraction of dietary fiber from orange peel by-products

**Fig 1:**
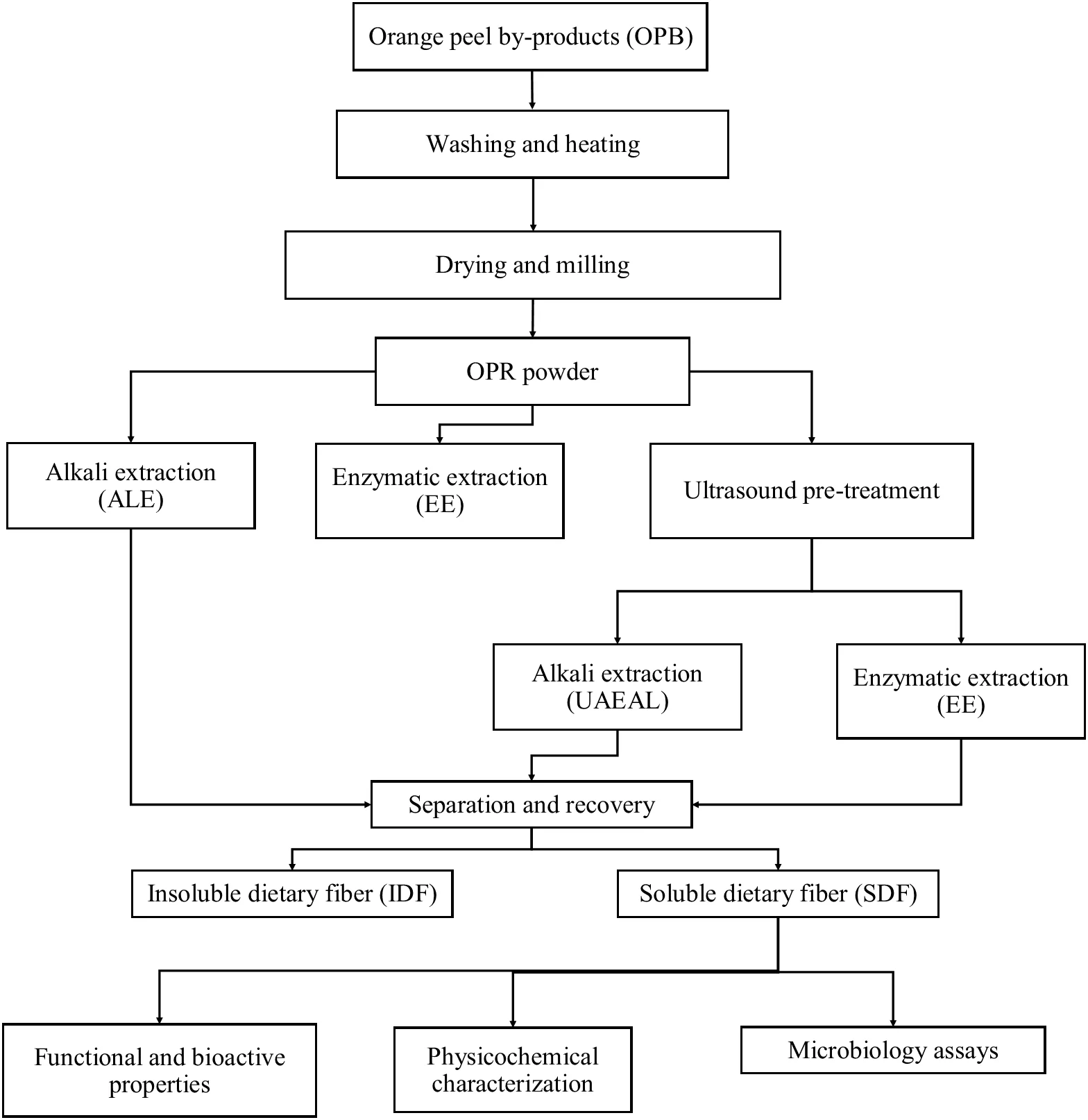
Flowsheet of the experimental protocol for extracting the SDF from OPB.

The extraction of soluble and insoluble dietary fibers was evaluated using methods based on those described by Kaur et al. [15], with some modifications. Fig 1 presents the flowsheet of the experimental extraction protocol and fiber characterization.

### Alkali extraction

For alkali extraction (ALE), 5 g of OPB powder was mixed with 1% (w/w) NaOH at a Solid-to-Liquid solvent-to-feed ratio of 25. The mixture was agitated at 500 rpm for 30 min at room temperature (25 °C). It was then incubated at 50 °C for 30 min in a thermostatic bath (VWR, WB05, Radnor, USA). Subsequently, the mixture was centrifuged (Hermle, Z326K, Wehingen, Germany) at 3620 g for 30 min to separate the residues from the supernatant. IDF was recovered from the wet pellet and dried in an oven at 45 °C for 24 hours. SDF was recovered from the supernatant by adding four volumes of 96% ethanol and incubating the mixture for 2 hours. Then, the residues were rinsed with 100% ethanol and dried in an oven under the same conditions as previously described. The extraction yield was calculated using Equation (1).


$$\begin{array}{c} Y(\%)=\frac{W_{f}}{Weight\;of\;OPR}*\text{1}\text{0}\text{0} \end{array}$$ (1)

Where *Y* denotes the yield of the recovered dietary fiber fraction (SDF or IDF), *W*_*f*_ denotes the dry weight of the recovered fiber, and *Weight of OPB* denotes the dry weight of the orange residues. TDF was calculated as the sum of the SDF and IDF weights.

### Enzymatic extraction

Enzymatic extraction was performed following the methodology described by Kaur et al. [15], with some modifications. 5 g of powdered OPB was weighed and suspended in 125 mL of 0.08 M phosphate buffer (pH 6.0). Subsequently, 100 µL of 1 M calcium chloride (CaCl_2_) was added, and the pH was adjusted to 6.0. Then, 0.25 mL of thermostable alpha-amylase (12000 U g^-1^) was added, and the mixture was incubated in a water bath at 90°C for 15 min. After the mixture was brought to room temperature, the pH was adjusted to 7.5 using NaOH, and 0.50 mL of protease (2.4 U g^-1^) was added, followed by incubation at 60°C for 30 min. The mixture was brought to room temperature, and the pH was adjusted to 4.5 with HCl. Subsequently, 1 mL of amyloglucosidase (104000 U g^-1^) was added. The mixture was incubated again at 60°C for 30 min. Finally, the mixture was cooled to room temperature, and the pH was adjusted to 6.5–7.0 to stop enzymatic activity. The sample was centrifuged under the same conditions used for alkaline treatment to recover SDF and IDF, as described earlier.

### Ultrasound pretreatment

A 750-W ultrasonic homogenizer (Cole-Parmer, 750W, Vernon Hills, USA) was used for ultrasound pretreatment. Five grams of orange peel powder were mixed with the appropriate solvent. After preliminary trials, ultrasound pretreatment conditions were set to pulse mode (2 seconds on, 2 seconds off) for 15 min at 60% amplitude and room temperature (25 °C). SDF and IDF were recovered using the previously described methodology for alkali and enzymatic extractions. UAEAL corresponds to alkali extraction with ultrasound pretreatment, whereas UAEE corresponds to the enzymatic extraction with ultrasound pretreatment.

### Determination of total dietary fiber content

Total dietary fiber content was determined using a total dietary fiber assay kit (TDF100A, Sigma-Aldrich) according to the AOAC 991.43 method. This method is based on a combination of enzymatic digestion and gravimetric determination. Dried samples were gelatinized with alpha-amylase, then digested with protease and amyloglucosidase to remove proteins and starches. Ethanol was then added to precipitate the SDF fraction. The resulting residue was filtered and washed sequentially with ethanol and acetone. After drying, the residue was weighed. Half of the samples were analyzed for protein content using the Kjeldahl method, while the remaining half were incinerated. The total dietary fiber content was calculated as the residue weight minus protein and ash weights.

### Functional and bioactive properties

Extraction of bioactive compounds from SDF was performed as described by Sheng et al. [16], with modifications. One gram of sample was suspended in 50 mL of water and placed in a thermostatic bath (Quimis, Q226M1, Diadema, Brazil) with constant agitation at 45 °C for one hour. The extraction solution was then separated from the insoluble residues by centrifugation (Quimis, Q222T108, Diadema, Brazil) at 3000 g for 5 min.

### Determination of total phenolic content

Total phenolic content was determined by the Folin–Ciocalteu method, using gallic acid as the standard, following the procedure of Ferreira-Anta et al. [17] with minor modifications. Briefly, 0.5 mL of the sample was mixed with 4 mL of deionized water and 0.5 mL of the Folin-Ciocalteu reagent. After 3 min of incubation, 0.5 mL of sodium carbonate solution (prepared by dissolving 1g of Na_2_CO_3_ in 3.5 mL of water and incubating for 30 min at 37°C) was added. Absorbance was measured at 660 nm using a spectrophotometer (Shimadzu UV-3600 Plus, Shimadzu, Kyoto, Japan). Results were expressed as milligrams of gallic acid equivalents per gram of biomass (mg GAE g^-1^).

### Determination of total flavonoid content

Total flavonoid content was determined via the aluminum chloride assay with catechin as the standard [18]. A 1 mL sample aliquot was mixed with 5 mL of ethanol (80% v/v) and 1 mL of NaNO_2_ solution (5% w/v) and allowed to react for 6 min. Subsequently, 1 mL of AlCl_3_ solution (10% w/v) was added and mixed thoroughly. After an additional 6 min, 10 mL of NaOH solution (1 M) was added. The mixture was then allowed to stand for 15 min before measuring the absorbance at 510 nm. The values were expressed as milligrams of catechin equivalents per gram of biomass (mg CE g^-1^).

### Water and oil-holding capacities

Water and oil-holding capacities were determined following the method described by Panswar et al. [12], with modifications. Water-holding capacity (WHC) was prepared by hydrating the powdered sample (0.5 g) with distilled water (10 mL) and vigorously mixing for 5 min. Then, it was allowed to rest for 30 min at room temperature; furthermore, it was centrifuged at 3620 g for 20 min. Finally, the supernatant was discarded, and the water holding capacity was calculated using Equation (2).


$$\begin{array}{c} WHC\;\left({g\;g}^{-\text{1}}\right)=\frac{W_{\text{2}}-W_{\text{1}}}{W_{\text{1}}} \end{array}$$ (2)

Where *W*_*1*_ is the weight of the sample, and *W*_*2*_ is the final weight of the sample.

Oil holding capacity (OHC) was determined by vigorously mixing a sample (0.5 g) with olive oil (10 mL) and whisking for 5 min. The mixture was kept at room temperature for 30 min, then centrifuged at 3620 g for 20 min. The supernatant was discarded, and the OHC was determined using equation (3):


$$\begin{array}{c} OHC\;\left({g\;g}^{-\text{1}}\right)=\frac{O_{\text{2}}-O_{\text{1}}}{O_{\text{1}}} \end{array}$$ (3)

Where O_1_ is the weight of the sample; O_2_ is the final weight of the sample.

### Fourier transform infrared (FTIR) spectroscopy

Infrared spectra were obtained using a spectrophotometer (PerkinElmer, Spectrum 400, Shelton, USA). Analytical-grade KBr pellets were used for measurements, with a resolution of 4 cm^-1^, a scanning range of 4000–600 cm^-1^, and 20 scan cycles.

### X-ray diffraction

The crystallinity of SDF was analyzed by X-ray diffraction using a diffractometer (Shimadzu, XRD-6000, Tokyo, Japan) equipped with a monochromatic graphite source and CuKα radiation, operating at 40 kV and 30 mA, with a scanning speed of 4°·min^-1^ and a 2θ scanning range of 10° to 80°. The degree of crystallinity was evaluated using the crystallinity index, calculated according to equation (4) described by de Sun et al. [19].


$$\begin{array}{c} Crystallinity\;index\;(\%)=\;\frac{I_{\text{0}\text{0}\text{2}}-\;I_{am}}{I_{\text{0}\text{0}\text{2}}}*\text{1}\text{0}\text{0} \end{array}$$ (4)

where *I*_*002*_ is the diffraction peak intensity of the crystalline material near 2θ = 20°, due to the nature of the soluble fiber, and *I*_*am*_ is the diffraction peak intensity of the amorphous material near 2θ = 15°. The values obtained from the crystallinity index are relative measures of molecular ordering in the polymer matrix.

### Scanning electron microscopy

Scanning electron microscopy was performed using a Hitachi S-3400N (Tokyo, Japan). Samples were mounted on carbon tape and coated with platinum. Images were obtained at an accelerating voltage of 10 kV and a magnification of ×500.

### Thermogravimetry analysis

The thermogravimetric analysis was performed using a thermogravimetric analyzer (Shimadzu, DTG-60H, Tokyo, Japan). 10 mg of the sample was weighed and placed in an alumina tray under a nitrogen atmosphere. The temperature was increased from 25 °C to 600 °C at a heating rate of 10 °C min^-1^, with a constant nitrogen flow of 50 mL min^-1^. An empty alumina tray was used as the reference.

### Microbiology assays

*Faecalibaculum rodentium* ABL288 isolate (IATA-CSIC internal microbial collection) and *Bacteroides tethaiotaomicron* DSM2079^T^ –obtained from DSMZ-German Collection of Microorganisms and Cell Cultures (Germany)- were propagated from frozen glycerol stock cultures. The *F. rodentium* strain was grown overnight in MRS modified medium prepared as follows: 10 g L^-1^ tryptone, 10 g L^-1^ meat extract, 5 g L^-1^ yeast extract, 5 g L^-1^ sodium acetate, 2 g L^-1^ di-ammonium citrate, 0.2 g L^-1^ magnesium sulfate, 0.05 g L^-1^ manganese sulfate, 0.5 g L^-1^ di-potassium hydrogen phosphate, 0.1% v/v polysorbate 80, and 0.05% (w/v) L -cysteine. The *B. tethaiotaomicron* was grown overnight in Wilkins-Chalgren (WC) modified medium prepared as follows: 10 g L^-1^ tryptone, 10 g L^-1^ meat extract, 10 g L^-1^ gelatin peptone, 5 g L^-1^ yeast extract, 5 g L^-1^ sodium chloride, 1 g L^-1^ L-arginine, 1 g L^-1^ sodium pyruvate, 5 mg L^-1^ menadione, and 5 mg L^-1^ hemin. For initial propagation, 0.5% (w/v) glucose was set as the main carbon source. Cultures were incubated at 37 °C for 48 h in an anaerobic jar with AnaeroGenTM bags (Thermo Fisher). Three passages of overnight cultures were diluted 1:20 into fresh, pre-warmed, oxygen-depleted media before testing different culture conditions. In all cases, Gram staining and Sanger sequencing of the 16S rRNA gene were performed on end-point cultures to discard cross-contamination. To test the capacity of OPB-derived soluble fiber to provide a substrate for *F. Rodentium* and *B. thetaiotaomicron* growth, 48 h cultures were diluted 1:20 in pre-warmed, oxygen-depleted medium, and OD600 was measured after 48 h of culture under strict anaerobic conditions. The experiment consisted of five independent replicates, and respective blanks were used to subtract the medium background and determine the specific absorbance by bacterial biomass. Growth rate was referred to the maximum signal obtained from MRS + Glc treatment.

### Statistical analysis

All data are presented as mean ± standard deviation (SD). Independent experiments were performed in triplicate (n = 3), except for water-holding capacity (WHC) and oil-holding capacity (OHC) measurements, which were conducted in duplicate (n = 2). The assumptions of normality, homogeneity of variances, and independence were verified prior to the analysis. Statistical significance was evaluated using one-way ANOVA followed by Tukey´s post hoc multiple comparisons test at a significance level of p < 0.05, in SPSS version 25. In all tables and figures, the presence of different lowercase letters indicates significant differences between treatment means. The Shapiro-Wilk test was used to assess data normality, and consequently, t-tests or Wilcoxon rank-sum tests were used to assess differences in data distributions for growth rates. A Bonferroni correction was used when multiple pairwise comparisons were evaluated simultaneously.

## Results and discussion

### Extraction yields

Table 1 presents the extraction yields of SDF and IDF from OPB across the treatments. SDF extraction yields ranged from 11.38 ± 0.50% to 26.04 ± 0.81% with ALE and UAEE, respectively. In contrast, IDF extraction yields ranged from 10.89 ± 0.63% to 23.51 ± 2.42% with UAEAL and UAEE. The extraction method significantly influences the extraction yield (p < 0.05). In this study, the EE method yielded higher results than the ALE method. For example, the extraction yields obtained with EE are double those obtained with ALE. The increase in yield observed with enzymatic treatment is associated with its ability to hydrolyze glycosidic bonds in insoluble components, such as cellulose and high-molecular-weight hemicellulose, thereby enhancing the conversion of IDF to SDF [20]. Moreover, the enzymatic method may promote the degradation or hydrolysis of polysaccharide chains by exposing more hydrophilic groups, yielding soluble oligosaccharides and β-dextrin. Enzymes can also degrade non-fibrous components, such as proteins, lipids, and starches, thereby increasing product purity. Furthermore, enzymatic hydrolysis increases substrate porosity and solvent penetration, facilitating the extraction of bioactive compounds [21].

**Table 1:** SDF, IDF, total dietary fiber content, and SDF purity obtained from OPB using different extraction treatments.

TREATMENTS	SDF (%)	IDF (%)	Total dietary fiber content (SDF + IDF, %)	SDF purity (%)
**ALE**	11.38 ± 0.50^d^	14.37 ± 0.09^bc^	25.75 ± 0.55^c^	85.51 ± 0.20^b^
**UAEAL**	14.27 ± 0.74^c^	10.89 ± 0.63^c^	25.16 ± 0.12^c^	86.61 ± 0.10^b^
**EE**	23.14 ± 0.59^b^	18.40 ± 0.23^b^	41.55 ± 0.23^b^	92.15 ± 0.54^a^
**UAEE**	26.04 ± 0.81^a^	23.51 ± 2.42^a^	49.55 ± 2.42^a^	91.09 ± 0.19^a^

Fiber extraction was done using four different treatments: ALE, EE, UAEAL, and UAEE. Different letters in the same column indicate significant differences between treatments using Tukey’s test (p < 0.05). Total dietary fiber was calculated as the sum of the SDF and IDF fractions, whereas SDF purity was calculated using the AOAC 991.43 method, which analyzed only the SDF fraction recovered after extraction. Purity values were corrected for residual protein and ash content.

The use of ultrasound as a pretreatment increased SDF yield by 12.5% and 25.0% for the EE and ALE methods, respectively. This enhancement is likely attributable to ultrasound-induced cavitation, which increases internal cell pressure, leading to cell wall disruption and facilitating the release of bioactive compounds [22]. By facilitating the mass transfer of solute through a higher surface area, these conditions enhance alkaline oxidation and enzymatic hydrolysis of the lignocellulolytic matrix [15]. Moreover, the intense shear forces and mechanical effects generated by acoustic cavitation, particularly the rapid collapse of transient bubbles, can disrupt the plant cell wall matrix and partially depolymerize insoluble polysaccharides. These effects may break both covalent and non-covalent interactions, such as hydrogen bonds, that maintain the structural organization of pectin and hemicellulose within the insoluble fiber network, thereby facilitating the conversion of IDF into SDF [23]. Based on preliminary standardization assays, the ultrasound probe pretreatment was applied under fixed operating conditions to ensure process reproducibility while minimizing excessive polymer degradation. These effects highlight the high efficiency of UAE as a pre-treatment for SDF recovery from plant matrices. Overall, the UAE achieves higher yields and improved mass transfer than conventional and non-ultrasound-assisted techniques. Conversely, in the ALE extraction method, the IDF yield decreased by 28% compared with the ultrasound method. Similar to the findings reported by Kaur et al. [24] for the extraction of pearl millet bran fiber using ALE and UAEAL, certain extraction conditions may dissolve cellulose and hemicellulose, converting IDF into SDF. This conversion increases SDF yield while decreasing IDF. In this study, ultrasound likely triggered this effect in the ALE treatment. In contrast, the EE treatment increased IDF yield by approximately 28%, similar to SDF, resulting in higher fiber recovery. This outcome is linked to the high selectivity of enzymatic hydrolysis, which specifically targets non-fibrous macromolecules (starch, proteins) without degrading or solubilizing cell wall components excessively.

An amplitude of 60% provided the acoustic energy required to disrupt the rigid cellulose lattice without inducing excessive thermal degradation that can occur at higher energy levels. Similarly, the 1:25 Liquid solvent-to-feed ratio allows mass transfer while preventing acoustic attenuation. As a result, the SDF yield obtained in this work was higher than those reported in recent citrus studies, such as Panwar et al. [12] (12.19%), Gu et al. [25] (10.56%), and Kaur et al. [26] (7.5%). However, to further improve this process, future research should focus on standardizing industrial-scale processes to ensure consistent fiber quality at lower operational costs.

The SDF yields obtained using EE and UAEE were not significantly different from those reported by Khanpit et al. [27]. In their study, SDF recovery from orange (*Citrus reticulata*) was evaluated using ultrasound and extrusion technologies, achieving yield increases of 22.27% at 400 W and 24.28% at 35°C. Similarly, the results of the present study were consistent with those reported by Kaur et al. [15], who obtained SDF yields of 12.45% and 24.47% using UAE and UAEE on kinnow by-products. In the present work, the highest SDF yield (26.04 ± 0.81) was achieved with UAEE, which is comparable to or higher than those reported by Zhou et al. [28] in SDF recovery from navel orange using deep eutectic solvents (DES) and ultrasound (26.00% − 22.11%).

Additionally, the purity percentage of SDF obtained through different treatments was evaluated (Table 1). The purity of SDF from OPB ranged from 85.51 ± 0.20% to 92.15 ± 0.54% for ALE and EE, respectively. Ultrasound use did not result in statistically significant changes in SDF purity. The EE allows for the hydrolysis of starch, depolymerizes high-molecular-weight proteins, and increases the production of low-molecular-weight proteins, which are easier to eliminate. Additionally, partial hydrolysis of cellulose and other structural polysaccharides promotes cell wall breakdown, facilitating the release of cellular content and the conversion of non-fibrous polymers into low-molecular-weight molecules [29]. The results indicate that enzymatic treatment is more effective at recovering a higher amount of high-purity SDF.

### Functional and bioactive properties

Table 2 shows the values of the phenolic compounds, which ranged from 3.23 ± 0.03 mg GAE g^-1^ to 12.15 ± 0.21 mg GAE g^-1^ for TPC, and from 2.15 ± 0.11 mg CE g^-1^ to 3.07 ± 0.08 mg CE g^-1^ for TFC. The SDF obtained via EE exhibited a higher content of bound bioactive compounds than ALE. This increased content is directly related to the extraction conditions, as enzymes selectively hydrolyze cellulose and cell wall structures while preserving functional compounds bound to the SDF matrix. In contrast, alkaline treatment (NaOH) promotes the degradation of phenolic compounds under harsh extraction conditions. Furthermore, ultrasound application not only enhanced extraction yields but also increased the content of bioactive compounds bound to SDF. A significant increase in TPC and TFC was observed following UAE pretreatment in both ALE and EE, with the highest concentrations of both compounds detected in the UAEE treatment. The increase in phenolic content observed after the UAE process can be attributed to ultrasonic cavitation affecting the cell wall structure and the insoluble fractions of the plant material. These waves exert strong disruptive forces on cellular tissues and membranes, thereby enhancing the release of bioactive compounds bound to SDF in both ALE and EE treatments [30]. As phenolic compounds are known for their antioxidant, anti-inflammatory, and anticancer activities, SDF from OPB can have several potential applications. For example, in the development of functional foods, primarily as a source of antioxidants that contribute to biological functions [31].

**Table 2:** Total phenolic content (TPC) and total flavonoids (TFC) of SDF extracted from OPB.

SDF	mg g^-1^ TPC	mg g^-1^ TFC
**ALE**	3.23 ± 0.03^d^	2.15 ± 0.11^c^
**UAEAL**	5.95 ± 0.05^c^	2.66 ± 0.06^b^
**EE**	10.20 ± 0.19^b^	2.63 ± 0.05^b^
**UAEE**	12.15 ± 0.21^a^	3.07 ± 0.08^a^

Fiber extraction was done using four different treatments: ALE, EE, UAEAL and UAEE. Different letters within the same column indicate significant differences between treatments using Tukey’s test (p < 0.05).

The phenolic content in SDF exceeded the values reported by Wang et al. [32] for orange peel fiber. In that study, the TPC ranged from 4.34 ± 0.21 mg GAE 100 g^-1^ to 5.81 ± 0.26 mg GAE 100 g^-1^, corresponding to samples obtained by aqueous extraction and by fermentation with *Lactobacillus plantarum*, respectively. However, these results were lower than those reported by Pamwar et al. [12], who observed a TPC of 23.73 ± 0.78 mg GAE g^-1^ and 29.06 ± 0.89 mg GAE g^-1^ for SDF extracted from *Citrus limetta* peels using ALE and UAEE treatments, respectively. Overall, the content of functional compounds associated with SDF was influenced by the extraction method applied. However, it depends not only on extraction efficiency but also on the intrinsic characteristics of the plant material, including its botanical nature, maturity stage, and agroclimatic conditions.

### Water-holding capacity (WHC) and oil-holding capacity (OHC)

**Fig 2:**
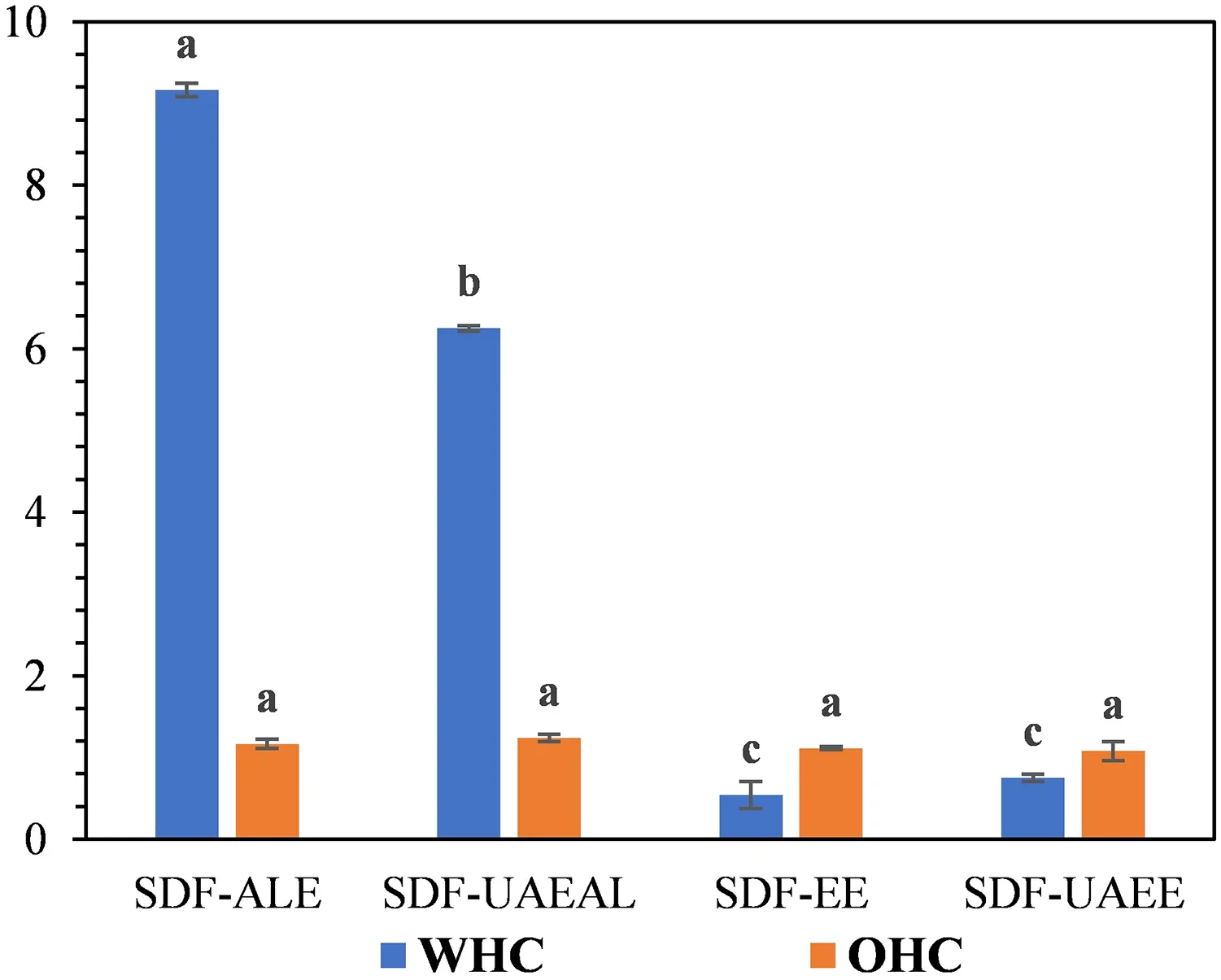
Water-holding capacity (WHC) and oil-holding capacity (OHC) of SDF. Bars with different lowercase letters indicate significant differences between treatments, as determined by Tukey´s test (p < 0.05).

The WHC and OHC values of different DF obtained from OPB are shown in Fig 2. Among the samples, ALE showed the highest WHC (ALE > UAEAL > UAEE > EE), which may be due to a greater abundance of hygroscopic oligomers or hydrophilic groups generated under alkaline conditions. These conditions facilitate water adsorption and retention by exposing active binding sites [33]. In contrast, the more disrupted structure observed in EE and UAEE treatments may explain their lower WHC values. Similar results were reported by Chen et al. [34] for DF obtained from white mulberry using UAEE. Structural reorganization may reduce water accessibility, leading to lower WHC in SDF extracted using EE than in SDF extracted using ALE. Likewise, Song et al. [35] reported that enzymatic modification led to degradation of polysaccharide networks, thereby negatively affecting WHC.

The hydration properties of SDF, reflected in its high WHC, are fundamental for food matrix design and intestinal health. High WHC is associated with a porous polymeric network and a high density of hydrophilic groups, which restricts the mobility of free water [36]. Technologically, this allows the SDF (particularly from ALE and UAEAL) to function as a natural thickening agent that reduces syneresis and improves the stability and shelf life of formulated foods. Physiologically, the viscous, gel-like structure of the SDF in the intestinal tract slows gastric emptying and small-intestinal transit, thereby increasing satiety and attenuating glucose spikes. Furthermore, these matrices bind bile acids and lipids, promoting their excretion and lowering total plasma cholesterol [37].

On the other hand, OHC values were low and did not differ significantly among SDF samples (p > 0.05), indicating that extraction treatments had little effect on OHC. This behavior may be attributed to the loss of hydrophobic binding sites and ester bonds within the polymeric structure during extraction [38]. The obtained OHC values are consistent with those of Zhang et al. [39], who reported OHC contents ranging from 1.15 ± 0.09 g g^-1^ to 1.40 ± 0.12 g g^-1^ for pawpaw fruit peel SDF. Given these functional properties, the SDF could have industrial applications. For instance, the fibers obtained through ALE and UAEAL are hypothesized to have applications in bakery and pastry products by promoting moisture retention and influencing freshness and shelf-life characteristics. This hypothesis is supported by studies showing that fiber integration can improve texture and moisture retention in baked goods [40,41]. Furthermore, the SDF fractions could potentially serve as fat replacers in dairy, meat, and emulsified food systems, although this remains to be experimentally validated in future studies. These properties suggest that the recovered fibers are promising candidates for functional food development, though further research is required to evaluate their performance in specific food systems.

### Fourier transform infrared (FTIR) spectroscopy

As can be observed in Table 3, all samples exhibit the characteristic adsorption bands of SDF components such as cellulose, hemicellulose, and pectin. Specifically, the presence of peaks near 2926–2929 cm^-1^ (C– H stretching in methylene groups) and 1595–1626 cm^^-1^^ (carbonyl groups) confirms that the core chemical identity of the orange peel remains intact regardless of the extraction method used [42]. Moreover, the shift in the hydroxyl group (-OH) from 3256 cm^-1^ (ALE) to 3275 cm^-1^ (UAEE) indicates a decrease in intermolecular hydrogen bonding. This suggests that the combined ultrasound pretreatment and enzymatic extraction effectively disrupted the plant matrix, making the fiber structure more porous compared with the conventional chemical extraction methods.

**Table 3:** FTIR spectral assignments and characteristic peak wavenumbers (cm^-1^) of SDF obtained from OPB using different treatments.

Functional group	Characteristic molecular vibration	ALE	UAEAL	EE	UAEE
Hydroxyl (−OH)	O–H stretching of hydroxyl groups associated with hemicellulose and pectin	3256	3275	3275	3275
Alkanes (C − H)	C-H stretching of methyl and methylene groups in polysaccharides	2929	2926	2928	2929
Carbonyl (C = O)	Stretching vibration of esterified carbonyl groups	1595	1595	1619	1626
Alkanes/ Acids (C − H / C − O)	Symmetric bending and stretching vibration of polysaccharides	1404	1406	1389	1395
Backbones (C − O − C / C − O)	Stretching vibration of saccharide units or piranose rings	1004	1013	1055	1061

**Fig 3:**
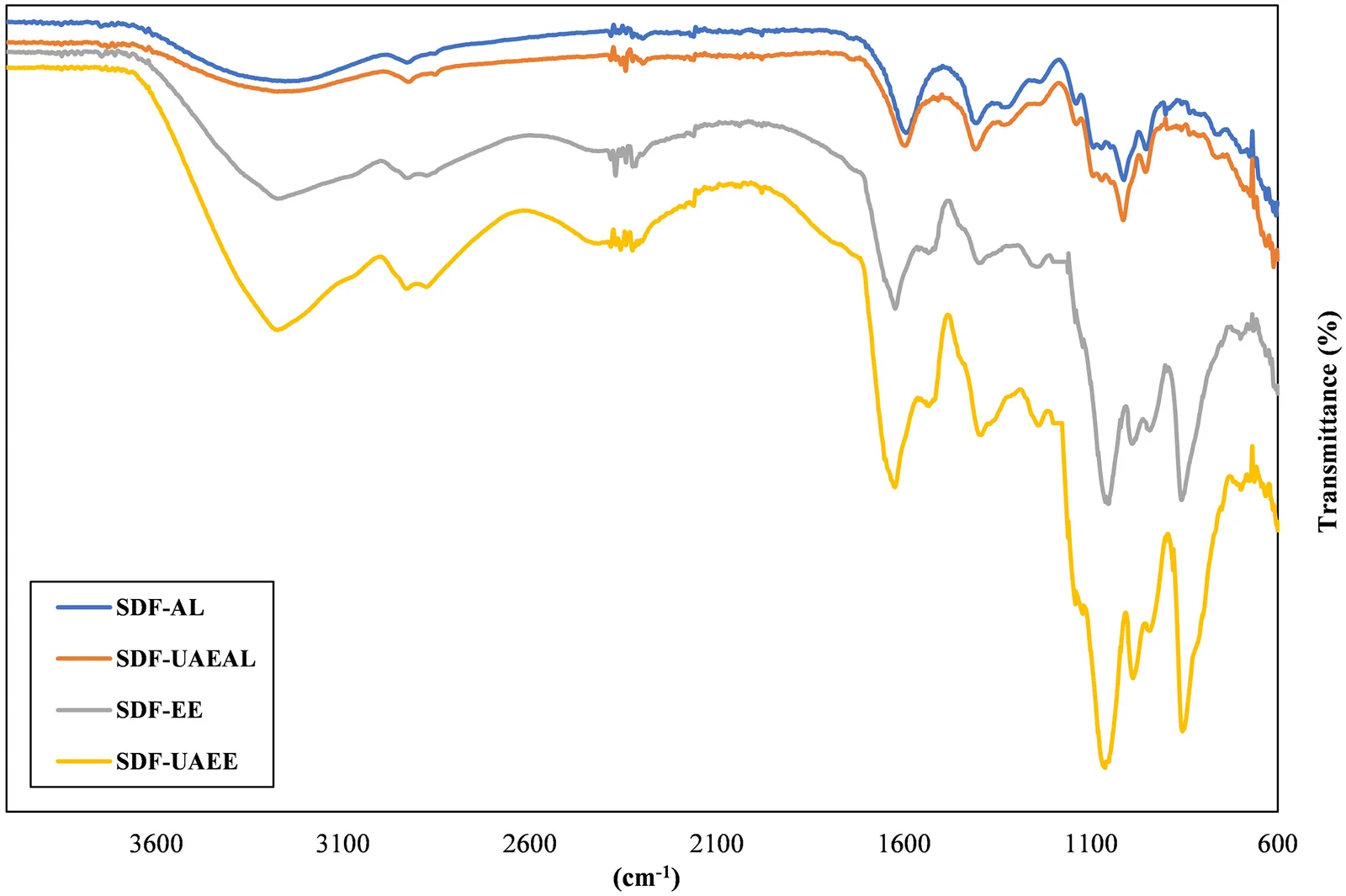
FTIR spectra of SDF obtained from OPB using different extraction treatments (ALE, UAEAL, EE, and UAEE).

The SDF obtained through ALE and EE treatments showed absorption spectra similar to those obtained through UAE-assisted extraction, as presented in Fig 3. Differences were identified in the intensity of characteristic bands between the alkaline and enzymatic treatments, mainly between 3200 cm^-1^ and 3300 cm^-1^, where the absorption band corresponding to the OH stretching vibration of hemicellulose and pectin was observed, as well as, the absorbed water distribution in the structure. This decrease in intensity in this spectral range could be attributed to the breaking of intermolecular bonds [21]. However, the OH wavenumber was lower in ALE than in EE, ranging from 3255 cm^^-1^^ to 3275 cm^-1^. Displacement to the blue indicates hydrogen bridge weakening in the polysaccharide matrix of SDF. This suggests that EE induces a major rupture of hydrogen-bonded intramolecular bonds in cellulose, thereby releasing more hydroxyl groups that vibrate. Whereas ALE consists of more ordered structures with a higher density of strong hydrogen bridges. Between 2923–2930 cm^-1^, the absorption of the stretching vibration of CH groups present in methyl or methylene form in polysaccharides was observed. These two bands indicate the presence of a typical molecular structure in polysaccharides, confirming the fibrous nature of SDF [43]. The absorption peaks observed between 1600 and 1622 cm^-1^ correspond to the C = O stretching vibration associated with uronic acid; their abundance and signal intensity indicate a high uronic acid content. In addition, the absorption interactions observed between 1200–1400 cm^-1^ are characteristic of saccharides derived from SDF in the FTIR spectrum, including CH bonds and their variable vibrational angles [25]. The absorption signal at approximately 1050 cm^-1^ is caused by the C–O stretching vibration of C–O–C in the sugar units of hemicellulose or the pyranose ring. This was observed at a lower intensity at 1026 cm^-1^ in the SDF obtained by UAEE from Citrus limetta [12]. The absorption intensities were relatively higher in the SDF obtained through the EE treatment. This is directly related to the higher content and density of glycosidic bonds in hemicellulose and pyranose rings, which are exposed during the extraction process. This may indicate that EE released these functional groups from the cellulose matrix, increasing their density in SDF and confirming the major presence of soluble polysaccharides in the final components [44]. On the other hand, in the spectral range of 700–1000 cm^-1^, peaks corresponding to the stretching vibration of β-glycosidic bonds in hemicellulose were identified, with greater intensity in UAEE at 857 cm^-1^ [45]. This result is consistent with the observations of Lin et al. [43], who identified the characteristic absorption peak of the β-glycosidic bond around 870 cm^-1^ in the SDF extracted from Shatian pomelo peel. This relates to the effects of ultrasound and enzymatic activity on hemicellulose and cellulose, which expose dipolar forms.

### X-ray diffraction analysis (XRD)

**Fig 4:**
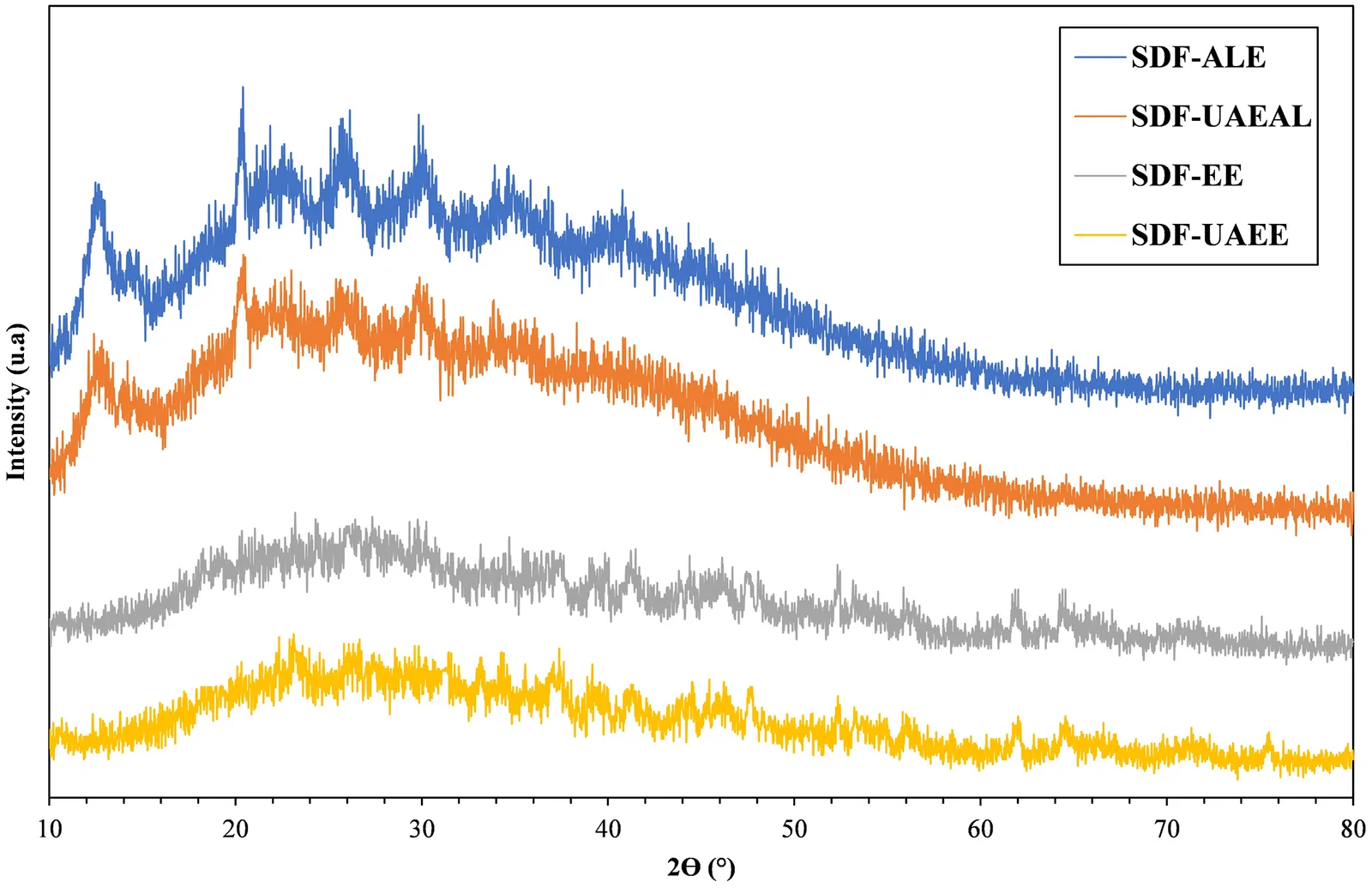
X-ray diffraction patterns of SDF obtained by different extraction treatments (ALE, UAEAL, EE, and UAEE).

XRD analysis was used to evaluate the crystallinity patterns of the SDF obtained from OPB (Fig 4). The diffraction patterns revealed clear differences among the different extraction treatments. SDF obtained by alkaline extraction exhibited characteristic cellulose peaks, although with lower intensity in UAEAL. Meanwhile, EE and UAEE treatments showed the highest amorphous fraction, indicating low crystallinity. The presence of amorphous regions is mainly associated with the content of hemicellulose and pectin. SDF obtained through ALE and UAEAL exhibited crystalline and semi-crystalline fractions, directly associated with the cellulose content and the massive dissolution of xylooligosaccharides during the alkaline process [7]. The diffraction patterns showed intense and regular peaks between 12.0° and 31.0°. The most intense signal was located at approximately 20.3°, characteristic of type I cellulose. The application of ultrasound during alkaline treatment reduced the crystallinity and diffraction peak intensity of SDF obtained by UAEAL, thereby weakening intermolecular interactions among SDF molecules [46]. Sonication induces rupture of glycosidic bonds (β-1–4), depolymerization, and alteration in crystalline cellulose structure. As observed in UAEAL, this depolymerization is more pronounced between 20.0° and 22.0°, where the main cellulose peak appears dampened and flattened. This result suggests that the crystalline structure suffered significant, but not total, degradation when ultrasound was applied, causing a structural transition from an ordered configuration to a less ordered one, indicating the conversion of crystalline structures to amorphous ones [39]. On the other hand, the SDF obtained via EE and UAEE exhibited low or no crystallinity, likely due to selective disruption and degradation of the cellulose structure by ultrasound and enzymatic hydrolysis. This means that the crystalline structure was disrupted, and the fiber remained uncrystallized after enzymatic treatment. The amorphous nature of EE- and UAEE-derived SDF was confirmed by the absence of cellulose peaks at 20.0°–22.0°, such as the peaks detected in ALE and UAEAL. Additionally, the intensity signal around 12.0° is practically absent. Consequently, the crystalline structure is transformed into an amorphous one, dissolving the cellulose and concentrating the hemicellulose and pectin.

**Fig 5:**
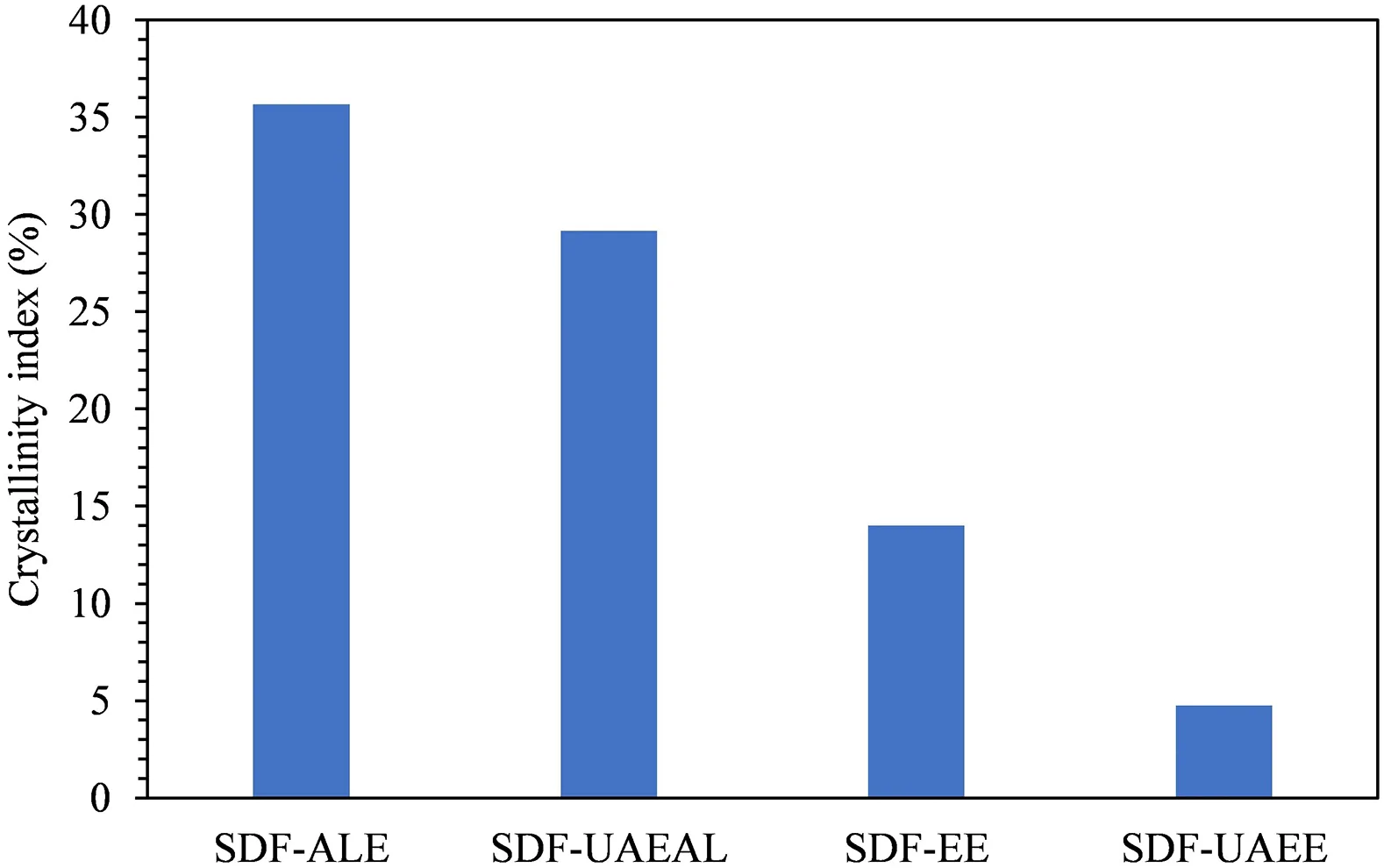
Crystallinity index of SDF obtained by different extraction treatments (ALE, UAEAL, EE, and UAEE).

As shown in Fig 5, the SDF obtained by ALE has the highest crystallinity index (35.67%), whereas the SDF obtained by UAEE has the lowest (4.76%). Alkaline or conventional treatments generally yield structures with higher crystalline or semicrystalline fractions because they cannot disrupt the compact packing of cellulose microfibrils or degrade certain amorphous components [38]. In contrast, the synergistic effect of ultrasonic pretreatment and enzymatic hydrolysis reduced the crystallinity index to a minimum value of 4.76%. This decrease further confirms the effect of ultrasonic waves on the polymeric network, facilitating enzymatic hydrolysis and overcoming the physical limitations of traditional extraction methods [12].

The decrease in the crystallinity index among the treatments was mainly attributed to the broadening of the crystalline peak around 20.4° and the absence of cellulose peaks at 20.0°–22.0°, confirming the impact of ultrasound on the crystalline lattice. These results align with those reported by Yang et al. [45], who found that ultrasonic waves weakened intermolecular interactions and reduced the crystallinity index from 23.28% to 17.65% after ultrasound treatment of SDF obtained from grapefruit peels. A similar effect was reported by Zhou et al. [28]. They found that the crystallinity index of SDF obtained using deep eutectic solvents combined with ultrasonic pretreatment decreased from 31.48% to 29.73% after ultrasound treatment, possibly due to disruption of cellulose chains by ultrasonic cavitation.

### Scanning electron microscopy

**Fig 6:**
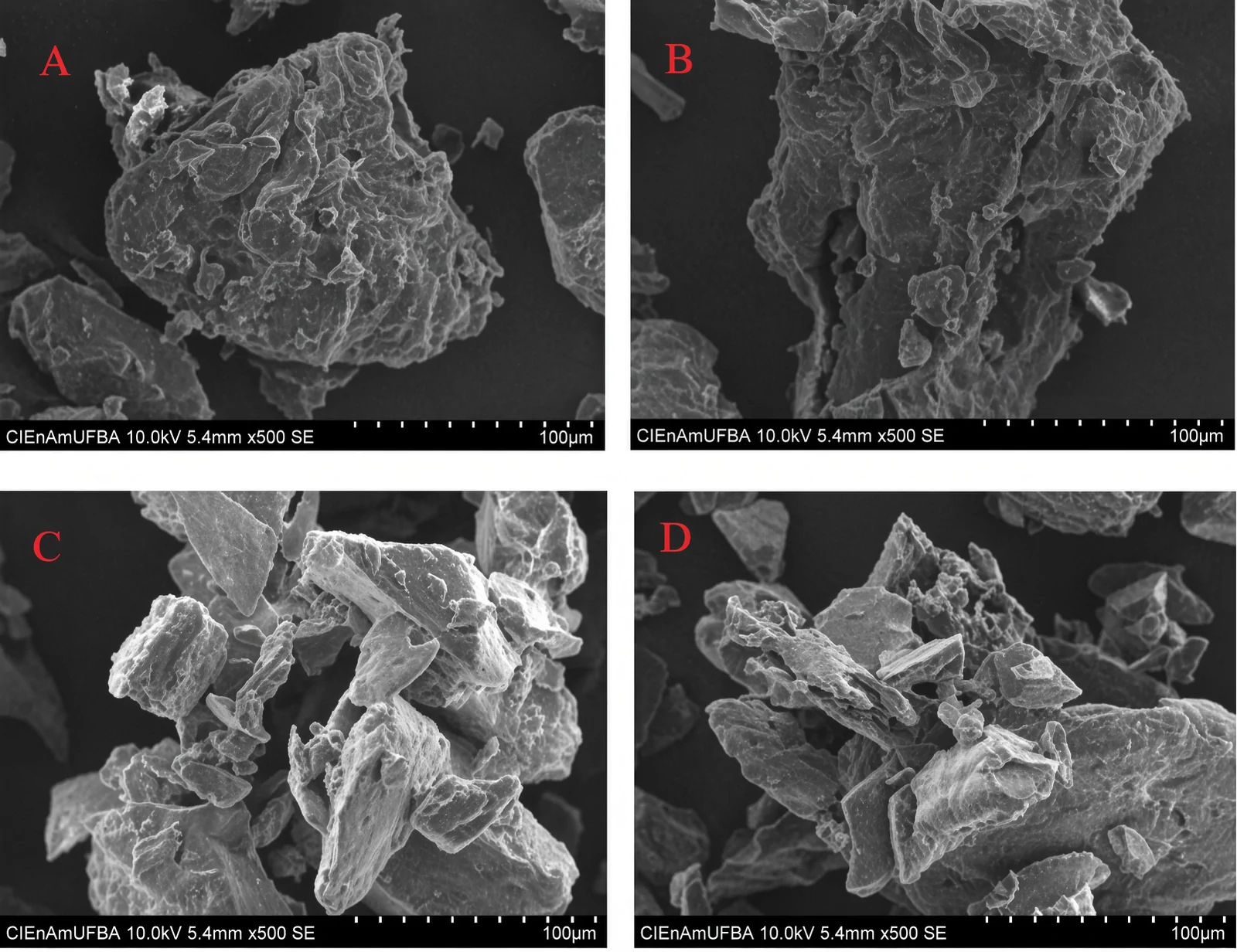
Scanning electron microscopy (SEM) images of SDF obtained by:ALE (A), EE (B), UAEAL (C), and UAEE (D).

The morphology of the SDF is shown in Figs 6A, 4B, 4C, and 4D. The Fig 6A corresponds to ALE, which exhibits an irregular, rough, and laminar surface structure. Alkaline extraction is characterized by the destructive action of sodium hydroxide on the cellular wall, primarily degrading hemicellulose while preserving cellulose fractions, as evidenced by X-ray diffraction patterns. Fig 6B corresponds to UAEAL, where greater disintegration is observed than in ALE. The application of ultrasound results in greater fragmentation and erosion of the plant material, leading to increased release of SDF and particles with a more exposed internal structure. Fig 6C and 6D correspond to EE and UAEE, which exhibit a finer, more granular, and more porous surface with a structure that tends to flatten and lose rigidity. Enzymatic hydrolysis acts on glycosidic bonds, disrupting the cell wall, reducing macromolecular weight, and producing a porous structure. These results are associated with a greater yield of SDF extraction, as reported by Li et al. [47]. Increasing porosity in EE and UAEE suggests greater hydration capacity; however, they showed the lowest values among the samples (Fig 2). This paradox is attributed to excessive structural decomposition of the fiber and the accumulation of low-molecular-weight fragments on the surface. These small molecules reduce surface area, limiting water-holding capacity [38].

As shown in Fig 6, the matrix appears smoother and more fragmented, indicating a high degree of depolymerization. This morphology also suggests possible interactions or crosslinking between SDF molecules, resulting from massive fragmentation induced by the combined action of ultrasound and enzymatic hydrolysis. The results demonstrate that ultrasound and enzymatic treatments transform the overall microstructure of SDF to a disordered or more degraded status. As shown in Figs 6C and 6D, the EE and UAEE samples exhibit smoother surfaces and a more disintegrated morphology with greater homogeneity, in contrast to the rough, fissured textures observed with alkali treatment. This transformation in microstructure is mainly due to differences in the degree of hydrolysis of cellulose, hemicellulose, and pectin, leading to more efficient depolymerization and smaller particle size.

Based on the morphological analysis, the macrostructural modifications induced by the different treatments directly affected the techno-functional properties of the resulting SDF. The implementation of ultrasonic pretreatment resulted in greater surface erosion and porosity in UAEAL, whereas UAEE exhibited a smoother and more compact surface due to greater structural disruption, excessive enzymatic hydrolysis, and the removal of protein components and starch [48]. Previous studies have reported that ultrasonic pretreatment is an innovative approach in which shock waves generated during cavitation act on the plant matrix, increasing surface roughness and promoting the formation of microcracks [49]. However, the morphological analysis revealed that this physical treatment may lead to bidirectional changes in SDF properties. Greater structural disruption and excessive cell fragmentation promote the accumulation of low-molecular-weight polymers on the surface, causing physical collapse that reduces the effective surface area, thereby limiting hydrophilicity and decreasing water retention capacity. Thus, the microscopic analysis revealed that innovative extraction techniques can modify the structural properties of SDF and demonstrated a critical processing threshold beyond which extreme microstructural disintegration redefines the matrix’s functional behavior [50].

As mentioned earlier, the TPC and TFC contents increased with the extraction treatments in the following order: ALE < UAEAL < EE < UAEE. Higher levels of these compounds were associated with increased antioxidant capacity through a structure–function relationship, in which microstructural and molecular modifications of SDF determine the accessibility and availability of bioactive compounds. Morphological analysis revealed the mechanical effects of ultrasonic pretreatment, whereby acoustic cavitation generates high pressures and shear forces that disrupt the compact matrix of orange residues, resulting in an SDF surface that is highly eroded and characterized by visible cracks and pores. This microstructural disruption of the matrix eliminates the steric hindrance imposed by the cell structure, facilitating mass transfer and the efficient release of phenolic compounds [50]. Meanwhile, the XRD patterns confirmed that ultrasonic pretreatment partially disrupted the intramolecular hydrogen bonds, leading to a disordered SDF matrix with a greater proportion of amorphous regions. This amorphous structure and the associated structural disruption reduced diffusion resistance, thereby maximizing the release and stabilization of phenolic compounds on the SDF surface [51].

### Thermogravimetry analysis

**Fig 7:**
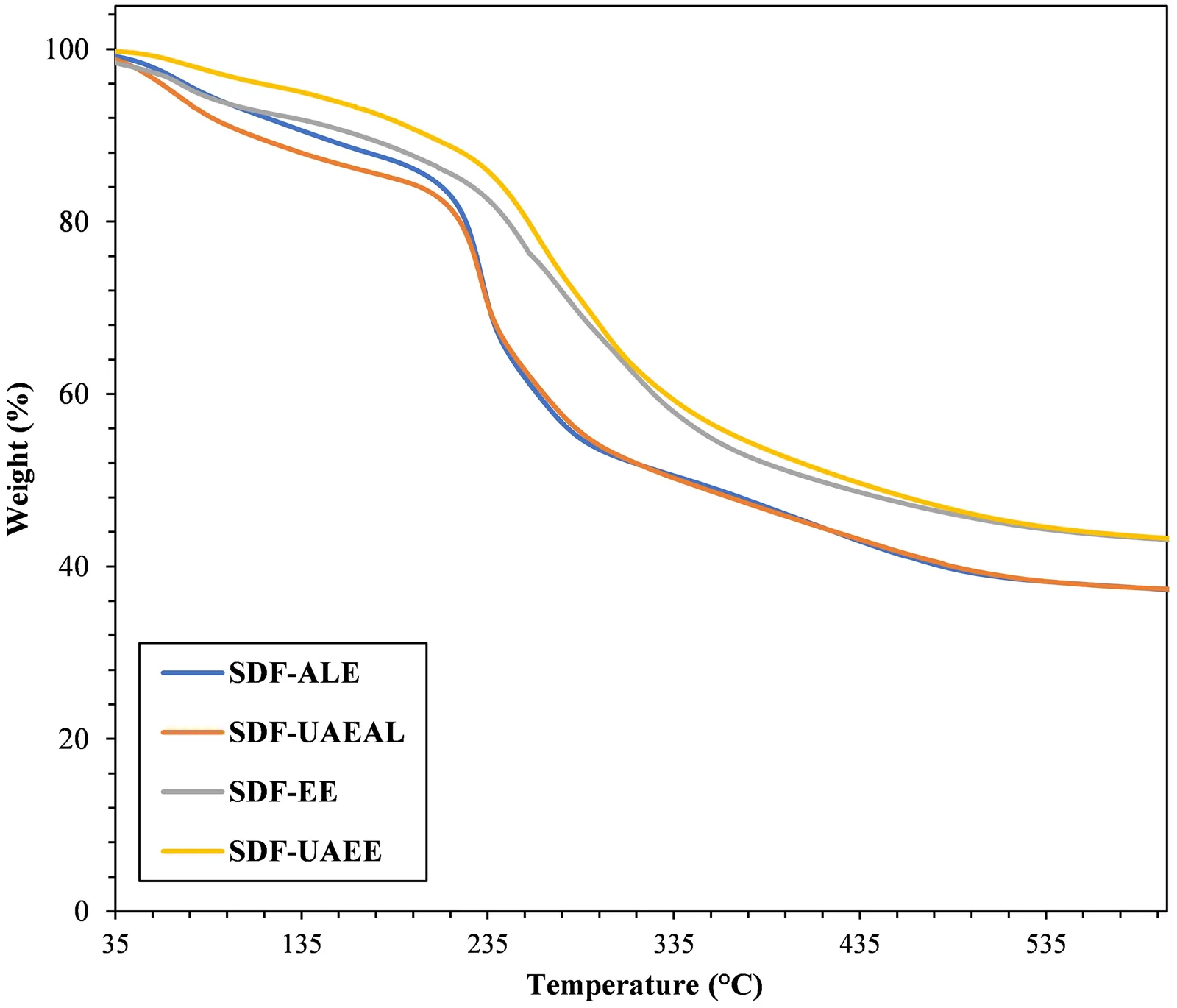
Thermogravimetry analysis curves of SDF obtained from OPB using different extraction treatments (ALE, UAEAL, EE, and UAEE).

The thermal stability of the SDF obtained from OPB was studied by thermogravimetric analysis, as shown in Fig 7. The SDF exhibited a similar behavior, divided into three stages. In the first stage, the temperature ranged from 35 to 200 °C, and the samples’ weight decreased slightly, likely due to the evaporation of bound and free water, resulting in weight losses of 9.72%−16.12% for UAEE and UAEAL, respectively. The second stage ranged from 200 to 400 °C; during this range, a significant mass decrease was observed. This weight loss is attributed to the gradual depolymerization of polysaccharides, which produces simultaneous cleavage of carbon-carbon (C-C) and carbon-oxygen (C-O) bonds in pyranose rings, as well as hydrogen-bond breakdown. This thermal decomposition process breaks down the monomeric unit, releasing volatile compounds such as carbon monoxide (CO), carbon dioxide (CO_2_), and water vapor. [52]. The mass loss of ALE and UAEAL was significantly higher, with values close to 54.51%. On the other hand, the fibers obtained by EE and UAEE showed a notably lower decrease, of approximately 48.46%. This difference is directly related to the pyrolytic decomposition of low–molecular–weight polysaccharides, which are more susceptible to thermal degradation [7]. The final mass percentage ranged from 37.34% to 43.26%, values corresponding to ALE and UAEE, respectively. UAEE exhibited greater thermal stability, as evidenced by a higher final residue percentage, whereas ALE showed less stable thermal properties. This finding indicates that combining ultrasound and enzymes yields an SDF with a molecular structure that is more resistant to thermal degradation. The profiles obtained for OPB-derived SDF exhibited characteristic thermal degradation patterns commonly reported for citrus-derived fibers. Based on the observed thermal behavior, SDF exhibited significant thermal stability at moderate temperatures. The results indicated the loss of volatile compounds and bound water from the SDF matrix at temperatures below 220 °C, suggesting that the structural components remain functionally intact after thermal treatment. The thermal resistance of SDF enhances its potential application in thermally processed food products, as it can be subjected to conventional processes such as drying, pasteurization, sterilization, and extrusion without compromising its functional or structural integrity [26]. Consequently, the observed thermal stability demonstrates that SDF derived from OPB could be used as a functional ingredient in moderate-temperature industrial processing applications, remaining below the depolymerization temperature of its structural polysaccharides.

### Microbiology assays

**Fig 8:**
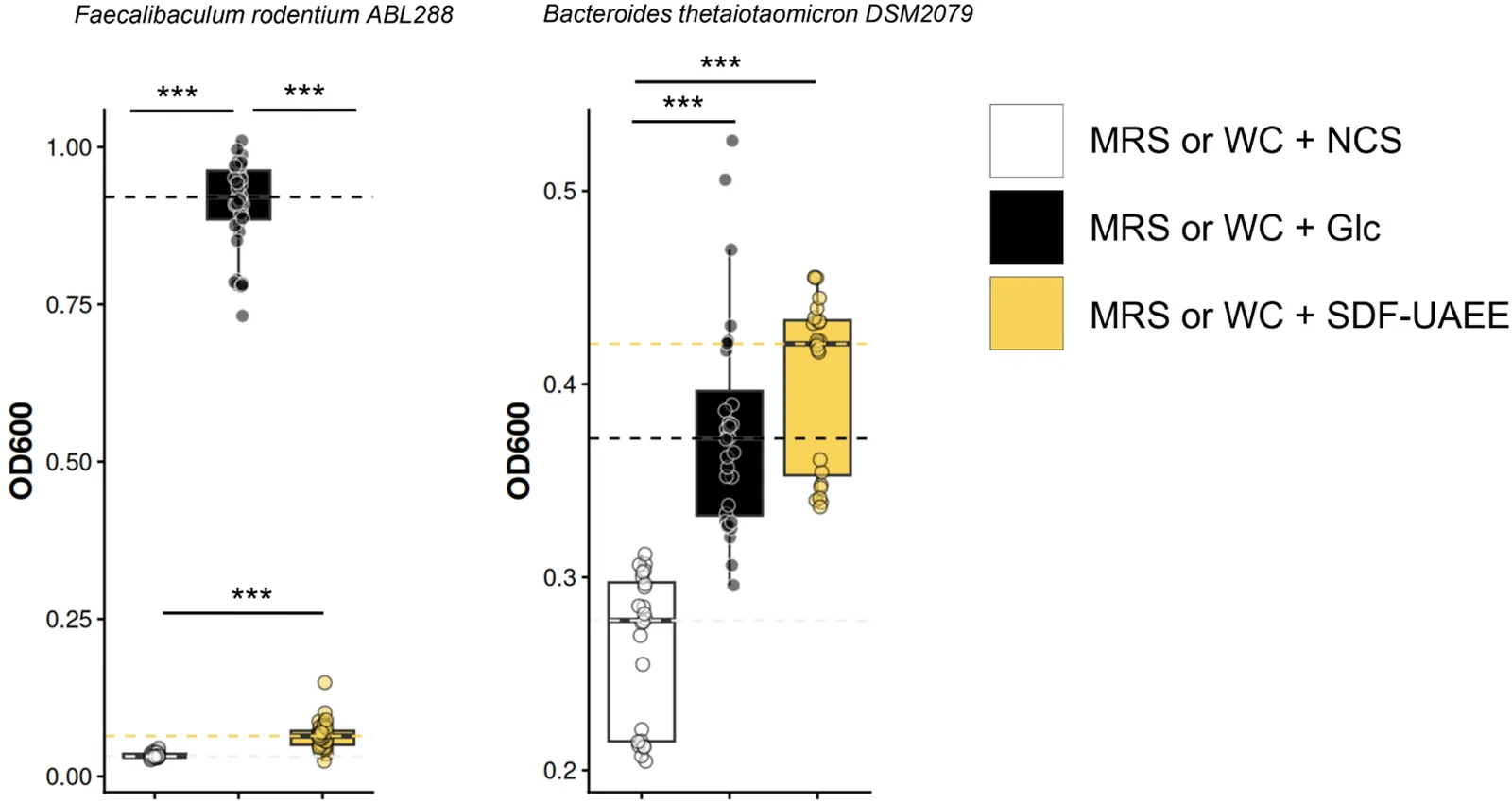
Microbiology assay with the OPB-derived soluble fiber. Biomass determination (OD600) after 48 h cultures in MRS supplemented with glucose (black) or SDF-UAEE (yellow) at 0.5% (w/v) for *Faecalibaculum rodentium* ABL288 or in WC supplemented with glucose (black) or SDF-UAEE (yellow) at 0.5% (w/v) for *Bacteroides thetaiotaomicron* DSM2079. Endpoint OD600 readouts were subtracted from respective media blanks cultured in similar conditions with no bacterial inoculum. MRS/WC + NCS, MRS or WC supplemented with no carbon source. Colored dashed lines indicate medians of respective OD600 distributions.

*F. rodentium* ABL288 and *B. thetaiotaomicron* DSM2079 were cultured in MRS and WC media, respectively, supplemented or not with a carbon source. We set three different conditions by modifying the media containing a carbon source: MRS/WC + Glc, MRS/WC + SDF-UAEE (autoclaved with fiber), and MRS/WC + NCS, containing glucose (0.5% w/v), OPB-derived soluble fiber (0.5% w/v), and no carbon source, respectively. As expected, *F. rodentium* exhibited a large growth rate in MRS supplemented with 0.5% glucose. In MRS supplemented with 0.5% SDF-UAEE, *F. rodentium* exhibited a modest growth rate, accumulating approximately 6.9% of the bacterial biomass compared with glucose-containing cultures, even though showing a significant growth rate increase with respect to the medium lacking a carbon source (6.9% vs 3.4%, p = 4.1E-12) (Fig 8). On the other hand, *B. thetaiotaomicron* showed better SDF-UAEE utilization performance. Although not significantly (p-adj > 0.05), it reached a median of 113%, the growth rate observed in the glucose condition, also indicative of substrate utilisation. The presence of glucose or SDF-UAEE in the medium represents a significant factor for *B. thetaiotaomicron* growth (p-adj = 6.6E-13) (Fig 6).

Seeking novel sources of dietary fiber with prebiotic properties is a major focus in food science. Such an aim becomes more prominent when it is embedded within the waste valorization and circular economy frameworks. Despite the impact of waste-derived dietary fibers being poorly tested in vivo, in vitro assessments indicate they could exert a pivotal impact on human and animal nutrition by promoting the growth of beneficial bacterial species, such as former Lactobacillus species and bifidobacteria [53,54]. Here, we tested the ability of the OPB soluble fiber to serve as a carbon source for the propagation of mammal gastrointestinal symbionts *Faecalibaculum rodentium* and *Bacteroides thetaiotaomicron* species with beneficial properties for host health [55]. Both symbionts showed substrate utilization signals ranging from modest to prominent. In the case of *F. rodentium*, although the growth rate did not reach the levels observed in the glucose-supplemented medium, the OPB soluble fiber appears to be a possible substrate for growth, as its growth pattern differs significantly from that in the medium with no carbon substrate. Notwithstanding, the metabolic potential of the OPB soluble fiber on this symbiont remains to be determined in future studies. We have previously evaluated the potential of several simple and complex fibers for other beneficial microbes, and large metabolic impacts with potential health claims do not necessarily encompass accelerated growth rates [56]. In the case of *B. thetaiotaomicron*, the growth rate was consistent with its recognized versatility for glycan utilization [57–59], reaching over 113% of the growth rate induced by glucose. Our results indicate that the OPB-derived soluble fiber obtained has the potential to impact the resident gastrointestinal microbial community of humans and other mammals, whether specialized or not in diet glycan utilization and degradation. Consequently, the impact of the product under consideration on humans, animals, and their gut microbes warrants further investigation into microbial metabolic outputs and their effects on host health.

## Conclusions

The study demonstrated that combining an ultrasound pretreatment with enzymatic hydrolysis (UAEE) yields the highest SDF recovery from OPB (26.04 ± 0.81%), surpassing conventional alkaline (ALE) and enzymatic (EE) methods. UAEE also delivers a highly pure SDF (91.09 ± 0.19%) and the highest levels of phenolic compounds (12.15 ± 0.21 mg GAE g-1) and flavonoids (3.07 ± 0.08 mg GAE g-1), indicating strong antioxidant potential. Functional analysis reveals that UAEE-derived SFD shows superior thermal stability and a marked amorphous structure, as confirmed by FTIR, XRD, and SEM. Aside from the appealing physicochemical properties of the fiber derived from OPB valorization, microbiological assays confirm that UAEE-SDF can serve as a carbon source for beneficial gut microbes, notably *Bacteroides thetaiotaomicron*, suggesting probiotic activity. The genomic response and metabolic output of microbes, and their impact on host health, while representing a limitation of our study, will constitute a promising area for future investigation.

## Abbreviations

ALE: Alkali extraction
DF: Dietary fiber
EE: Enzymatic extraction
FTIR: Fourier transform infrared spectroscopy
GAE: Gallic acid equivalents
IDF: Insoluble dietary fiber
OHC: Oil-holding capacity
OPB: Orange peel by-products
SDF: Soluble dietary fiber
SEM: Scanning electron microscopy
TDF: Total dietary fiber
TFC: Total flavonoid content
TGA: Thermogravimetry analysis
TPC: Total phenolic content
UAEAL: Alkali extraction with ultrasound pretreatment
UAEE: Ultrasound-pretreated enzymatic extraction
WHC: Water-holding capacity
XRD: X-ray diffraction.
