# Supplementary material for: Valorization of orange peel by-products via ultrasound-pretreated enzymatic extraction of soluble dietary fiber: Structural and functional characterization
Source: PLoS One. 2026 Sep 1;21(9):e0357376. doi: 10.1371/journal.pone.0357376 (PMC13533398; doi:10.1371/journal.pone.0357376)
Supplement: Supplementary file 1 [file pone.0357376.s001.docx]

Review Comments to the Author:

Editor: The reply and manuscript’s text adjustments to the Editor´s comments were highlighted in yellow. We thank the editor for the thorough review of the manuscript.

• Novelty: Explicitly state what is new beyond applying known ultrasound and enzyme extraction to orange peel. Cite distinguishing factors in the final Introduction paragraph.

Reply: We added the following text to the final Introduction paragraph.

Moreover, evidence on the application of ultrasound as a pretreatment for SDF recovery from orange residues at low mass-to-volume ratios, such as 1:25, and under temperature-controlled sonication conditions remains limited. This mass-to-volume ratio offers a critical advantage by reducing solvent consumption and minimizing waste generation, thereby facilitating process scalability and industrial implementation. Beyond these operational advantages, the innovative contribution of this work lies in transcending the conventional physicochemical characterization by validating the biological functionality of the recovered SDF.

• Objective scope: Rewrite the objective to cover all treatments, including alkaline, ultrasound-alkaline, enzymatic, and UAEE, as well as all measured endpoints, including IDF, TDF, functional properties, structural properties, thermal properties, and bacterial growth.

Reply: We have modified the objective.

This study aims to evaluate the effects of different extraction methods (alkaline extraction, alkaline extraction with ultrasonic pretreatment, enzymatic extraction, and enzymatic extraction with ultrasonic pretreatment) on the extraction yield of dietary fiber fractions from OPB. The influence of these treatments on the techno-functional and structural properties of SFD was also assessed. Additionally, its potential as a carbon source for the growth of *Faecalibaculum rodentium* ABL288 was evaluated.

• Yield equation: Fix Equation (1) by providing a complete formula with denominator, define all terms, and specify whether the calculation is based on dry weight.

Reply: We checked Equation (1).

• Extraction conditions: Justify fixed ultrasound and enzymatic parameters, including 60% amplitude, pulse mode, temperature, and pH. State whether these were optimized, literature-based, or derived from preliminary trials.

Reply: Enzymatic extraction was performed following the methodology described by Kaur et al. [17], with some modifications. Ultrasound pretreatment conditions were derived from preliminary trials. This information was added to the text.

• Controls: Add untreated OPB, ultrasound-only, and enzyme-free buffer controls, or provide a clear justification for their omission.

Reply: We did not include untreated orange peel residues and enzyme-free buffer because the starting material underwent a preliminary treatment to remove low-molecular-weight sugars, organic acids, and non-fibrous soluble materials. Consequently, a buffer-only control would have provided additional data on the solubilization of the complex polysaccharide network that constitutes dietary fiber, since these compounds had already been eliminated. Moreover, the ultrasound-only control was omitted because, in this work, ultrasound was evaluated exclusively as a pretreatment to enhance mass transfer until the alkaline or enzymatic extraction processes. This approach aligns with the literature, which indicates that ultrasound typically enhances chemical or biological processes, thereby increasing fiber extraction.

• TDF reporting: Clarify whether total dietary fiber is analytically measured using AOAC 991.43 or calculated as SDF + IDF. Revise the column heading accordingly.

Reply: In Table 1, total dietary fiber was calculated as the sum of SDF and IDF. SDF purity was measured analytically using AOAC 991.43. For this analysis, only the SDF fraction was analyzed with the Sigma-Aldrich Total Dietary Fiber Assay Kit to determine SDF purity as the ratio of SDF to the SDF measured by AOAC 991.43. We have revised the column heading and modified the text to clarify this.

• SDF purity: Present the full SDF purity dataset, including the reported 85.51 to 92.15% range, in a table and include protein and ash corrections if applicable.

Reply: We added this information to the table.

• Replicates: Report biological and technical replicates explicitly for every assay, not only for the microbiology experiments.

Reply: The following information was added to the text.

All experiments were conducted in triplicate, except for WHC and OHC measurements, which were performed in duplicate.

• Statistics: Specify n-values, define whether data are presented as mean ± SD or mean ± SE, confirm ANOVA assumptions, and clarify letter groupings in all tables and figures.

Reply: We have clarified these parameters in the revised manuscript. All data are presented as mean ± standard deviation (SD). Independent experiments were performed in triplicate (n = 3), with the exception of water holding capacity (WHC) and oil holding capacity (OHC) measurements, which were conducted in duplicate (n = 2). The assumptions of normality, homogeneity of variances, and independence were verified prior to the analysis. Statistical significance was evaluated using one-way ANOVA followed by Tukey´s post hoc multiple comparisons test at a significance level of p < 0.05, in SPSS version 25. In all tables and figures, the presence of different lowercase letters indicates significant differences between treatment means.

• Units: Standardize all units, including rpm versus g, decimal and comma notation, and enzyme activity, according to SI units and journal style.

Reply: We have checked all units. However, we maintained the unit rpm (500 rpm) exclusively for agitation (stirring), as this refers to the rotational speed of the magnetic stirrer rather than a centrifugal separation force.

• Carbon-source inconsistency: Resolve conflicting glucose and SDF concentrations, 0.05% versus 0.5% w/v, in the MRS media description.

Reply: We acknowledge the reviewer's point and apologize for the inconsistency. We have harmonized the value describing the substrate concentration (0.5 g/L w/v) throughout the text.

• Positive control: Include a recognized prebiotic control, such as inulin, FOS, or GOS, for valid B. longum growth comparison, or substantially weaken the prebiotic claims.

Reply: We would like to notify you that, after a comprehensive review of our records regarding the culture setup and traceability, we have found that the mislabeling in the samples produced data shown in the original submission does not correspond to *Bifidobacterium longum* ABL182 cultures but rather to *Faecalibaculum rodentium* ABL288 cultures. *F. rodentium* is a mammal gastrointestinal symbiont belonging to the *Erysipelothricaceae* family and is closely related to *Holdemanella biformis* (doi: 10.1007/s10482-015-0583-3), a quite relevant human symbiont we have previously characterized (doi: 10.1096/fj.202100126R). We have corroborated via molecular markers (16S rRNA gene sequencing) that the culture was pure, and we have retained the correctly identified data and added new replicates that are consistent with previous data. Moreover, we added novel information on the culture conditions of a mammal and human symbiont, recognized as a paradigm of glycan utilization versatility, *Bacteroides thetaiotaomicron* (doi: 10.1186/1471-2164-14-873). The response of *B. thetaiotaomicron* to OPB-derived fiber has been distinct from that of *F. rodentium*, confirming its high capacity for glycan utilization and showing better, though not significant, growth rates under glucose conditions. Consequently, we have modified the text to reflect this new configuration of microbiology assays, while toning down the meaning of our findings to avoid overclaiming (lines 206-224, 512-252, 539-555).

• Overclaiming: Revise the abstract, discussion, and conclusion because SDF-UAEE biomass of approximately 6.1% relative to glucose indicates limited substrate utilization rather than strong prebiotic potential.

Reply: We have toned down our overclaiming and aligned the conclusions with the scope of the results.

• Metabolic evidence: Supplement OD₆₀₀ data with SCFA production, pH reduction, or carbohydrate-consumption data. At minimum, acknowledge the lack of metabolic evidence as a key limitation.

Reply: Such limitation was explicitly stated in the conclusion section.

• TGA inconsistency: Correct whether thermogravimetric analysis was performed on SDF or IDF, and revise the section and figure caption consistently.

Reply: Thermogravimetric analysis was performed on SDF. This information was clarified in the text.

• XRD quantification: Calculate and report a crystallinity index or quantitative peak analysis instead of relying solely on visual interpretation.

Reply: We added the crystallinity index to the text using the following methodology:

The degree of crystallinity was evaluated using the crystallinity index, calculated according to equation (4) described by de Miranda et al. [25].

$$Crystallinity index (\%)= \frac{I_{002}- I_{am}}{I_{002}}*100$$

where I002 is the diffraction peak intensity of the crystalline material near 2θ = 20° due to the nature of the soluble fiber, and *I_am_* is the diffraction peak intensity of the amorphous material near 2θ = 15°. The values obtained from the crystallinity index are relative measures of molecular ordering in the polymer matrix.

Moreover, the following discussion was added in the results and discussion section:

As shown in Fig. 4, the SDF obtained by ALE has the highest crystallinity index (36%), whereas the SDF obtained by UAEE has the lowest (5%). The decrease in the crystallinity index among the treatments was mainly attributed to the broadening of the crystalline peak around 20.4° and the absence of cellulose peaks at 20.0°–22.0°, confirming the impact of ultrasound on the crystalline lattice. These results align with those reported by Yang et al. [68], who found that ultrasonic waves weakened intermolecular interactions and reduced the crystallinity index from 23.28% to 17.65% after ultrasound treatment of SDF obtained from grapefruit peels. A similar effect was reported by Zhou et al. [30]. They found that the crystallinity index of SDF obtained using deep eutectic solvents combined with ultrasonic pretreatment decreased from 31.48% to 29.73% after ultrasound treatment, possibly due to disruption of cellulose chains by ultrasonic cavitation.

• FTIR interpretation: Provide a table of major bands, assignments, and peak shifts or intensity changes for each treatment instead of relying on a purely descriptive narrative.

Reply: We added the values corresponding to the peak wavenumbers of the functional groups (cm-1) for the different treatments in Table 3 below:

As can be observed in Table 3, all samples exhibit the characteristic adsorption bands of SDF components such as cellulose, hemicellulose, and pectin. Specifically, the presence of peaks near 2926 - 2929 cm^-1^ (C– H stretching in methylene groups) and 1595 - 1626 cm^-1^ (carbonyl groups) confirms that the core chemical identity of the orange peel remains intact regardless of the extraction method used. Moreover, the shift in the hydroxyl group (-OH) from 3256 cm-1 (ALE) to 3275 cm-1 (UAEE) indicates a decrease in intermolecular hydrogen bonding. This suggests that the combined ultrasound pretreatment and enzymatic extraction effectively disrupted the plant matrix, making the fiber structure more porous compared with the conventional chemical extraction methods.

Table 3. FTIR spectral assignments and characteristic peak wavenumbers (cm⁻¹) of SDF obtained from OPB using different treatments.

| Functional group | Characteristic molecular vibration | ALE | UAEAL | EE | UAEE |
| --- | --- | --- | --- | --- | --- |
| Hydroxyl (−OH) | O–H stretching of hydroxyl groups associated with hemicellulose and pectin | 3256 | 3275 | 3275 | 3275 |
| Alkanes (C−H) | C-H stretching of methyl and methylene groups in polysaccharides | 2929 | 2926 | 2928 | 2929 |
| Carbonyl (C=O) | Stretching vibration of esterified carbonyl groups | 1595 | 1595 | 1619 | 1626 |
| Alkanes/ Acids (C−H / C−O) | Symmetric bending and stretching vibration of polysaccharides | 1404 | 1406 | 1389 | 1395 |
| Backbones (C−O−C / C−O) | Stretching vibration of saccharide units or piranose rings | 1004 | 1013 | 1055 | 1061 |

• Figure 4 caption: Correct SEM labels so that A to D correspond to ALE, UAEAL, EE, and UAEE, respectively, and fix all omissions and misalignments in the caption.

Reply: We checked the Figure caption.

• Industrial claims: Reframe food-fortification and bakery shelf-life statements as future hypotheses because no food-system experiments were conducted.

Reply: We have reframed the section as follows:

Given these functional properties, the SDF could have industrial applications. For instance, the fibers obtained through ALE and UAEAL are hypothesized to have applications in bakery and pastry products by promoting moisture retention and influencing freshness and shelf-life characteristics. This hypothesis is supported by studies showing that fiber integration can improve texture and moisture retention in baked goods [26, 46]. Furthermore, the SDF fractions could potentially serve as fat replacers in dairy, meat, and emulsified food systems, although this remains to be experimentally validated in future studies. These properties suggest that the recovered fibers are promising candidates for functional food development, though further research is required to evaluate their performance in specific food systems.

• Language and formatting: Submit the manuscript for professional English editing and correct grammar errors, inconsistent capitalization, and typographical errors, including “he application,” “micrography,” and “amorphous status.”.

Reply: We have checked the manuscript using Grammarly Pro and with a native English-speaking colleague. We hope the manuscript meets the requirements.

Reviewer #1: The reply and manuscript’s text adjustments to Reviewer #1's comments were highlighted in green. We thank the Reviewer for the thorough review of the manuscript.

Introduction: The introduction should be reviewed to cover the following key points and should relate them to current updated research. should be given regarding:

• The problem and its significance should be introduced in a way that reflects the current situation, helping readers understand the motivation behind the research and highlighting the importance of utilizing value-added materials.

Reply: Thank you for your comment. We have added the following text to improve the introduction.

Orange is one of the most important crops worldwide, with an estimated annual production of 47 million tons in 2024 [1]. Globally, the management of citrus residues is a critical environmental challenge, as millions of tons of organic waste are generated each year, releasing significant amounts of greenhouse gases, including carbon dioxide and methane, during decomposition [2]. These by-products, which include peel, albedo, and endocarps, account for up to 55% of the fruit's weight. In Colombia, where production reached 756 thousand tons in 2023, the common practice of discarding these residues in landfills represents a substantial loss of biomass rich in carotenoids, flavonoids, pectin, and dietary fiber (DF) [3]. Consequently, in accordance with the principles of the circular economy, it is imperative to develop valorization strategies that mitigate environmental impacts while recovering high-value functional ingredients [4].

• Detailed technical information on extraction methods should be included, along with introductory information on the research methods, to help readers understand the relevant innovations and technologies.

Reply: Thank you for the comment. We have added the following text to improve the introduction.

Traditional chemical extraction methods (acidic or alkaline) are common due to low cost but often require high temperatures and pose environmental challenges [12]. In contrast, ultrasound-assisted extraction (UAE) is a sustainable “green” technology that operates at lower temperatures with reduced solvent requirements [13]. The core mechanism is acoustic cavitation: sound waves (typically> 20 kHz) create microbubbles that grow and then collapse, releasing high-intensity energy. This generates hydrodynamic shear forces that induce cell wall disruption. This structural modification increases the surface area and improves mass transfer, allowing solvents and reagents to penetrate the internal structure more efficiently, thereby increasing the recovery of DF fractions [14, 15].

• Links to updated and current research should be included to highlight gaps in previous research and frame your study within a broader context.

Reply: Thank you for the comment. We have added the following text to improve the introduction.

Conventional techniques (such as acid, alkaline, and hot-water extraction) are often limited by low extraction yields and the risk of polysaccharide degradation from harsh reagents and high temperatures. For example, Bakr et al. [19] reported a modest SDF yield of 5.61% from quince using alkaline extraction. In contrast, green technologies such as ultrasound-assisted enzymatic extraction have demonstrated higher efficiency. Panwar et al. [18] achieved an SDF yield of 12.19% from sweet lime pomace using this approach. These alternative methods represent a significant innovation in fiber purity and preserving structural integrity while adhering to environmentally sustainable practices.

Materials and Methods

• The unit descriptions should be consistent throughout the article, and details of the research instruments used, such as model name, brand, city, and country of manufacture, should be provided.

Reply: Thanks for the comment. We have reviewed the unit descriptions and the instrument details.

• Extraction of dietary fiber from orange peel by-products: An infographic should be included to facilitate understanding for the reader.

Reply: Thank you for your comment. We have added the following flowsheet to the Materials and Methods section:

Figure. Flowsheet of the experimental protocol for extracting the SDF from OPR.

• More detailed explanations should be added regarding "Determination of total phenolic content" and "Determination of total flavonoid content".

Reply: Thank you for your comment. We have revised this section as follows:

**Determination of total phenolic content**

Total phenolic content was determined using the Folin–Ciocalteu method, with gallic acid as the standard, following the procedure described by Inocêncio et al. [20]. Briefly, 0.5 mL of the sample was mixed with 4 mL of deionized water and 0.5 mL of the Folin-Ciocalteu reagent. After 3 min of incubation, 0.5 mL of sodium carbonate solution (prepared by dissolving 1g of Na₂CO₃ in 3.5 mL of water and incubating for 30 min at 37°C) was added. Absorbance was measured at 660 nm using a spectrophotometer (Shimadzu UV-3600 Plus, Shimadzu, Kyoto, Japan). Results were expressed as milligrams of gallic acid equivalents per gram of biomass (mg GAE g⁻¹).

**Determination of total flavonoid content**

Total flavonoid content was determined via the aluminum chloride assay with catechin as the standard [21]. A 1 mL sample aliquot was mixed with 5 mL of ethanol (80% v/v) and 1 mL of NaNO₂ solution (5% w/v) and allowed to react for 6 min. Subsequently, 1 mL of AlCl₃ solution (10% w/v) was added and mixed thoroughly. After an additional 6 min, 10 mL of NaOH solution (1 M) was added. The mixture was then allowed to stand for 15 min before measuring the absorbance at 510 nm. The values were expressed as milligrams of catechin equivalents per gram of biomass (mg CE g⁻¹).

• A detailed explanation of the statistical analysis, including the experimental design and all the statistics used in this study, should be added.

Reply: We have checked and revised this section of the manuscript.

Results and Discussion: The writing style should be concise, unnecessary parts should be removed, and the focus should be on explanations that are consistent with the experimental results. Up-to-date and current references should be used.

• Add comparative information with other extraction methods to help readers see the advantages and limitations of the technique used in the research and to assess whether using this technique is more cost-effective.

Reply: Thank you for your comment. We have reviewed this section and expanded the discussion on this topic.

• Explain how ultrasound waves affect the extraction of dietary fiber from orange peels, helping readers understand the key mechanisms and how to improve the extraction process for greater efficiency.

Reply: Thanks for your comment. We have added the additional information to the text.

Moreover, the intense shear forces and mechanical effects generated by acoustic cavitation, particularly the rapid collapse of transient bubbles, can disrupt the plant cell wall matrix and partially depolymerize insoluble polysaccharides. These effects may break both covalent and non-covalent interactions, such as hydrogen bonds, that maintain the structural organization of pectin and hemicellulose within the insoluble fiber network, thereby facilitating the conversion of IDF into SDF [29].

• Analyze the various factors that affect the extraction results, helping readers understand the optimal conditions for extracting dietary fiber from orange peels and to improve the process in the future. Include a comprehensive and up-to-date comparison of the results of this research with previous studies.

Reply: Thank you for your comment. We have expanded the analysis of the factors.

An amplitude of 60% provided the acoustic energy required to disrupt the rigid cellulose lattice without inducing excessive thermal degradation that can occur at higher energy levels. Similarly, the 1:25 ratio allows mass transfer while preventing acoustic attenuation. As a result, the SDF yield obtained in this work was higher than those reported in recent citrus studies, such as Panwar et al. [16] (12.19%), Gu et al. [31] (10.56%), and Kaur et al. [32] (7.5%). However, to further improve this process, future research should focus on standardizing industrial-scale processes to ensure consistent fiber quality at lower operational costs.

• A more in-depth explanation of the functional properties of dietary fiber should be added. This will help readers understand the benefits of soluble dietary fiber in various fields.

Reply: Thank you for your comment. We have expanded the functional properties of dietary fiber.

The hydration properties of SDF, reflected in its high WHC, are fundamental for food matrix design and intestinal health. High WHC is associated with a porous polymeric network and a high density of hydrophilic groups, which restricts the mobility of free water. Technologically, this allows the SDF (particularly from ALE and UAEAL) to function as a natural thickening agent that reduces syneresis and improves the stability and shelf life of formulated foods. Physiologically, the viscous, gel-like structure of the SDF in the intestinal tract slows gastric emptying and small-intestinal transit, thereby increasing satiety and attenuating glucose spikes. Furthermore, these matrices bind bile acids and lipids, promoting their excretion and lowering total plasma cholesterol.

• Describe the changes that occur in the properties of dietary fiber obtained from extraction and compare this research with previous research to show readers how innovative techniques can improve dietary fiber properties.

Reply: Thank you for your comment. We have expanded the results and the section as outlined in the following paragraphs.

Alkaline or conventional treatments generally yield structures with higher crystalline or semicrystalline fractions because they cannot disrupt the compact packing of cellulose microfibrils or degrade certain amorphous components [61]. In contrast, the synergistic effect of ultrasonic pretreatment and enzymatic hydrolysis reduced the crystallinity index to a minimum value of 4.76%. This decrease further confirms the effect of ultrasonic waves on the polymeric network, facilitating enzymatic hydrolysis and overcoming the physical limitations of traditional extraction methods [18].

Based on the morphological analysis, the macrostructural modifications induced by the different treatments directly affected the techno-functional properties of the resulting SDF. The implementation of ultrasonic pretreatment resulted in greater surface erosion and porosity in UAEAL, whereas UAEE exhibited a smoother and more compact surface due to greater structural disruption, excessive enzymatic hydrolysis, and the removal of protein components and starch [77]. Previous studies have reported that ultrasonic pretreatment is an innovative approach in which shock waves generated during cavitation act on the plant matrix, increasing surface roughness and promoting the formation of microcracks [78]. However, the morphological analysis revealed that this physical treatment may lead to bidirectional changes in SDF properties. Greater structural disruption and excessive cell fragmentation promote the accumulation of low-molecular-weight polymers on the surface, causing physical collapse that reduces the effective surface area, thereby limiting hydrophilicity and decreasing water retention capacity. Thus, the microscopic analysis revealed that innovative extraction techniques can modify the structural properties of SDF and demonstrated a critical processing threshold beyond which extreme microstructural disintegration redefines the matrix's functional behavior [50].

• The impact of ultrasound extraction on increasing antioxidant capacity and an explanation of the relationship between structure and function should be included.

This will help highlight the optimization of extraction and the development of dietary fiber properties.

Reply: Thank you for your comment. We have added the following paragraph.

As mentioned earlier, the TPC and TFC contents increased with the extraction treatments in the following order: ALE < UAEAL < EE < UAEE. Higher levels of these compounds were associated with increased antioxidant capacity through a structure–function relationship, in which microstructural and molecular modifications of SDF determine the accessibility and availability of bioactive compounds. Morphological analysis revealed the mechanical effects of ultrasonic pretreatment, whereby acoustic cavitation generates high pressures and shear forces that disrupt the compact matrix of orange residues, resulting in an SDF surface that is highly eroded and characterized by visible cracks and pores. This microstructural disruption of the matrix eliminates the steric hindrance imposed by the cell structure, facilitating mass transfer and the efficient release of phenolic compounds [50]. Meanwhile, the XRD patterns confirmed that ultrasonic pretreatment partially disrupted the intramolecular hydrogen bonds, leading to a disordered SDF matrix with a greater proportion of amorphous regions. This amorphous structure and the associated structural disruption reduced diffusion resistance, thereby maximizing the release and stabilization of phenolic compounds on the SDF surface [51].

Conclusion

• The conclusion should be written in a concise format. Summarize the key research findings, explain the meaning of the study results, suggest directions for future research, and identify the limitations of the study. This will provide readers with an overview of the research and highlight areas for future development and application.

Reply: Thank you for your comment. We have rewritten the conclusions.

• Abbreviation: Should be added abbreviation section.

Reply: Thank you for your comment. We have added a list of abbreviations.

• References: The citation format should be reviewed according to the journal's requirements. Furthermore, the most up-to-date citations should be selected.

Reply: Thank you for your comment. We have checked the references and updated them.
