# Supplementary material for: Valorization of orange peel by-products via ultrasound-pretreated enzymatic extraction of soluble dietary fiber: Structural and functional characterization
Source: PLoS One. 2026 Sep 1;21(9):e0357376. doi: 10.1371/journal.pone.0357376 (PMC13533398; doi:10.1371/journal.pone.0357376)
Supplement: Supplementary file 2 [file pone.0357376.s002.docx]

Review Comments to the Author:

Editor: The reply and manuscript’s text adjustments to the Editor´s comments were highlighted in yellow. We thank the editor for the thorough review of the manuscript.

• The conclusions contain an important inconsistency. The manuscript states that enzymatic extraction represents the best extraction method due to its higher yield and purity, while later concluding that ultrasound-pretreated enzymatic extraction demonstrated the highest extraction yield and the most promising structural and functional properties. Please reconcile these statements and clearly identify the most suitable extraction method based on the overall results.

Reply: We agree with the Editor´s comments regarding the terminology. In the revised manuscript, we have clarified that the enzymatic methods (as a category) are superior to conventional alkaline extraction in terms of yield and purity; the ultrasound-pretreated enzymatic method is identified as the most suitable method overall. This is because UAEE achieved the significantly highest yield (26.04%), while maintaining a high purity level (91.09%) that is statistically comparable to EE treatment. Furthermore, UAEE demonstrated superior performance in TPC and TFP, and thermal stability, as now stated in the updated Conclusions section.

• Several statements regarding industrial applicability appear stronger than the experimental evidence supports. Claims such as “technical feasibility for industrial-scale application” and “strong potential for industrial application” should be moderated unless supported by pilot-scale, techno-economic, or scale-up data.

Reply: Thank you for your comment. We have moderated our claims to ensure they are strictly supported by our experimental evidence.

• The discussion of the prebiotic potential should be presented more cautiously. The current findings demonstrate that the recovered fiber can support the growth of Bifidobacterium longum under the tested conditions, but broader health, microbiome, or functional claims require additional metabolic, genomic, or in vivo evidence.

Reply: Thank you for your comment. We have moderated the discussion and conclusions to reflect the preliminary nature of these findings.

• Please improve several sentences in the Conclusions section that are difficult to follow grammatically. For example, expressions such as “transferring a greater amount of stabilizing capacity fibers” and “demonstrated the highest extraction yield and the most promising in structural and functional properties” should be revised for clarity and readability.

Reply: Thank you for your comment. We have rewritten the conclusions section.

• The statement that EE provides the highest yield should be carefully checked against the reported data showing that UAEE achieved the highest SDF recovery (26.04 ± 0.81%). The interpretation of yield versus purity should be clarified throughout the manuscript.

Reply: We thank the editor for pointing out this inconsistency. We have carefully checked our data and clarified the distinction between the general enzymatic approach and the optimized UAEE protocol.

• The manuscript frequently refers to enhanced antioxidant potential based on higher phenolic and flavonoid contents. Please ensure that all antioxidant-related statements are restricted to the measured parameters and do not imply biological activity beyond the available evidence.

Reply: We thank the editor for this clarification. We have updated all antioxidant-related statements to ensure they are strictly restricted to the measured in vitro parameters.

• Please consider adding a brief statement acknowledging that further studies are required to evaluate process scalability, economic feasibility, and performance in real food systems before industrial implementation.

Reply: Thank you for the comment. We have added the required statement at the end of the Conclusions section.

• A final thorough English language revision is recommended to improve sentence structure, reduce awkward phrasing, and enhance overall readability. While the scientific content is generally sound, linguistic polishing would significantly improve the manuscript.

Reply: We have completed the final language revision. We hope this time we meet the requirements.
