# Supplementary material for: Valorization of orange peel by-products via ultrasound-pretreated enzymatic extraction of soluble dietary fiber: Structural and functional characterization
Source: PLoS One. 2026 Sep 1;21(9):e0357376. doi: 10.1371/journal.pone.0357376 (PMC13533398; doi:10.1371/journal.pone.0357376)
Supplement: Peer review history

# Peer review: Decision Letter 0

**DOI:** 10.1371/journal.pone.0357376.r001

**Authors:** Karthikeyan Venkatachalam

9 Jun 2026

Dear Dr. Osorio-Tobón,

Thank you for submitting your manuscript to PLOS ONE. After careful consideration, we feel that it has merit but does not fully meet PLOS ONE’s publication criteria as it currently stands. Therefore, we invite you to submit a revised version of the manuscript that addresses the points raised during the review process.

Please submit your revised manuscript by Jul 24 2026 11:59PM. If you will need more time than this to complete your revisions, please reply to this message or contact the journal office at plosone@plos.org. When you're ready to submit your revision, log on to https://www.editorialmanager.com/pone/ and select the 'Submissions Needing Revision' folder to locate your manuscript file.

- A letter that responds to each point raised by the academic editor and reviewer(s). You should upload this letter as a separate file labeled 'Response to Reviewers'.
- A marked-up copy of your manuscript that highlights changes made to the original version. You should upload this as a separate file labeled 'Revised Manuscript with Track Changes'.
- An unmarked version of your revised paper without tracked changes. You should upload this as a separate file labeled 'Manuscript'.

If you would like to make changes to your financial disclosure, please include your updated statement in your cover letter. Guidelines for resubmitting your figure files are available below the reviewer comments at the end of this letter.

If applicable, we recommend that you deposit your laboratory protocols in protocols.io to enhance the reproducibility of your results. Protocols.io assigns your protocol its own identifier (DOI) so that it can be cited independently in the future. For instructions see: https://journals.plos.org/plosone/s/submission-guidelines#loc-laboratory-protocols. Additionally, PLOS ONE offers an option for publishing peer-reviewed Lab Protocol articles, which describe protocols hosted on protocols.io. Read more information on sharing protocols at https://plos.org/protocols?utm_medium=editorial-email&utm_source=authorletters&utm_campaign=protocols.

As the corresponding author, your ORCID iD is verified in the submission system and will appear in the published article. PLOS supports the use of ORCID, and we encourage all coauthors to register for an ORCID iD and use it as well. Please encourage your coauthors to verify their ORCID iD within the submission system before final acceptance, as unverified ORCID iDs will not appear in the published article. *Only*  the individual author can complete the verification step; PLOS staff *cannot*  verify ORCID iDs on behalf of authors.

We look forward to receiving your revised manuscript.

Kind regards,

Karthikeyan Venkatachalam, Ph.D.

Academic Editor

PLOS One

**Journal Requirements:**

When submitting your revision, we need you to address these additional requirements.

1. Please ensure that your manuscript meets PLOS ONE's style requirements, including those for file naming. The PLOS ONE style templates can be found at

https://journals.plos.org/plosone/s/file?id=wjVg/PLOSOne_formatting_sample_main_body.pdf and

https://journals.plos.org/plosone/s/file?id=ba62/PLOSOne_formatting_sample_title_authors_affiliations.pdf

2. Thank you for stating the following financial disclosure:

“Jhonatan D. Quiñonez-Ensuncho, Beatriz E. Valdés-Duque, and J. Felipe Osorio-Tobón are grateful to the Institución Universitaria Colegio Mayor de Antioquia for funding this work - Convocatoria 2020. Gala Rosales and Álvaro S. Lima thank the Coordenação de Aperfeiçoamento de Pessoal de Nível Superior - Brasil (CAPES) - Finance Code 001. Laura Ortega-Ruiz and Alfonso Benítez-Páez thank the Spanish Ministry of Science, Innovation and Universities (PID2023-150523NB-I00) for funding ABP. The Accreditation as Center of Excellence Severo Ochoa CEX2021-001189-S, funded by MICIU/AEI/10.13039/501100011033, is also fully acknowledged.”

Please state what role the funders took in the study.  If the funders had no role, please state: "The funders had no role in study design, data collection and analysis, decision to publish, or preparation of the manuscript."

If this statement is not correct you must amend it as needed.

Please include this amended Role of Funder statement in your cover letter; we will change the online submission form on your behalf.

3. Please note that your Data Availability Statement is currently missing the repository name and/or the DOI/accession number of each dataset. If your manuscript is accepted for publication, you will be asked to provide these details on a very short timeline. We therefore suggest that you provide this information now, though we will not hold up the peer review process if you are unable.

4. If the reviewer comments include a recommendation to cite specific previously published works, please review and evaluate these publications to determine whether they are relevant and should be cited. There is no requirement to cite these works unless the editor has indicated otherwise.

**Additional Editor Comments:**

Novelty: Explicitly state what is new beyond applying known ultrasound and enzyme extraction to orange peel. Cite distinguishing factors in the final Introduction paragraph.

Objective scope: Rewrite the objective to cover all treatments, including alkaline, ultrasound-alkaline, enzymatic, and UAEE, as well as all measured endpoints, including IDF, TDF, functional properties, structural properties, thermal properties, and bacterial growth.

Yield equation: Fix Equation (1) by providing a complete formula with denominator, define all terms, and specify whether the calculation is based on dry weight.

Extraction conditions: Justify fixed ultrasound and enzymatic parameters, including 60% amplitude, pulse mode, temperature, and pH. State whether these were optimized, literature-based, or derived from preliminary trials.

Controls: Add untreated OPB, ultrasound-only, and enzyme-free buffer controls, or provide a clear justification for their omission.

TDF reporting: Clarify whether total dietary fiber is analytically measured using AOAC 991.43 or calculated as SDF + IDF. Revise the column heading accordingly.

SDF purity: Present the full SDF purity dataset, including the reported 85.51 to 92.15% range, in a table and include protein and ash corrections if applicable.

Replicates: Report biological and technical replicates explicitly for every assay, not only for the microbiology experiments.

Statistics: Specify n-values, define whether data are presented as mean ± SD or mean ± SE, confirm ANOVA assumptions, and clarify letter groupings in all tables and figures.

Units: Standardize all units, including rpm versus g, decimal and comma notation, and enzyme activity, according to SI units and journal style.

Carbon-source inconsistency: Resolve conflicting glucose and SDF concentrations, 0.05% versus 0.5% w/v, in the MRS media description.

Positive control: Include a recognized prebiotic control, such as inulin, FOS, or GOS, for valid B. longum growth comparison, or substantially weaken the prebiotic claims.

Overclaiming: Revise the abstract, discussion, and conclusion because SDF-UAEE biomass of approximately 6.1% relative to glucose indicates limited substrate utilization rather than strong prebiotic potential.

Metabolic evidence: Supplement OD₆₀₀ data with SCFA production, pH reduction, or carbohydrate-consumption data. At minimum, acknowledge the lack of metabolic evidence as a key limitation.

TGA inconsistency: Correct whether thermogravimetric analysis was performed on SDF or IDF, and revise the section and figure caption consistently.

XRD quantification: Calculate and report a crystallinity index or quantitative peak analysis instead of relying solely on visual interpretation.

FTIR interpretation: Provide a table of major bands, assignments, and peak shifts or intensity changes for each treatment instead of relying on a purely descriptive narrative.

Figure 4 caption: Correct SEM labels so that A to D correspond to ALE, UAEAL, EE, and UAEE, respectively, and fix all omissions and misalignments in the caption.

Industrial claims: Reframe food-fortification and bakery shelf-life statements as future hypotheses because no food-system experiments were conducted.

Language and formatting: Submit the manuscript for professional English editing and correct grammar errors, inconsistent capitalization, and typographical errors, including “he application,” “micrography,” and “amorphous status.”

[Note: HTML markup is below. Please do not edit.]

Reviewers' comments:

Reviewer's Responses to Questions

**Comments to the Author**

1. Is the manuscript technically sound, and do the data support the conclusions?

Reviewer #1: Yes

**********

2. Has the statistical analysis been performed appropriately and rigorously?

Reviewer #1: Yes

**********

3. Have the authors made all data underlying the findings in their manuscript fully available?

The PLOS Data policy

Reviewer #1: Yes

**********

4. Is the manuscript presented in an intelligible fashion and written in standard English?

Reviewer #1: Yes

**********

Reviewer #1: This article discusses " Valorization of orange peel by-products via ultrasound-pretreated enzymatic extraction of soluble dietary fiber: structural and functional characterization ". It is useful information for readers interested in orange peel by-products. However, some aspects of the content should be improved to make the article completer and more credible to readers.

Introduction:

The introduction should be reviewed to cover the following key points and should relate them to current updated research. should be given regarding:

-The problem and its significance should be introduced in a way that reflects the current situation, helping readers understand the motivation behind the research and highlighting the importance of utilizing value-added materials.

-Detailed technical information on extraction methods should be included, along with introductory information on the research methods, to help readers understand the relevant innovations and technologies.

-Links to updated and current research should be included to highlight gaps in previous research and frame your study within a broader context.

Materials and Methods

-The unit descriptions should be consistent throughout the article, and details of the research instruments used, such as model name, brand, city, and country of manufacture, should be provided.

-Extraction of dietary fiber from orange peel by-products: An infographic should be included to facilitate understanding for the reader.

-More detailed explanations should be added regarding "Determination of total phenolic content" and "Determination of total flavonoid content".

-A detailed explanation of the statistical analysis, including the experimental design and all the statistics used in this study, should be added.

Results and Discussion

The writing style should be concise, unnecessary parts should be removed, and the focus should be on explanations that are consistent with the experimental results. Up-to-date and current references should be used.

Extraction yields

- Add comparative information with other extraction methods to help readers see the advantages and limitations of the technique used in the research and to assess whether using this technique is more cost-effective.

- Explain how ultrasound waves affect the extraction of dietary fiber from orange peels, helping readers understand the key mechanisms and how to improve the extraction process for greater efficiency.

- Analyze the various factors that affect the extraction results, helping readers understand the optimal conditions for extracting dietary fiber from orange peels and to improve the process in the future. Include a comprehensive and up-to-date comparison of the results of this research with previous studies.

Functional and bioactive properties

-A more in-depth explanation of the functional properties of dietary fiber should be added. This will help readers understand the benefits of soluble dietary fiber in various fields.

-Describe the changes that occur in the properties of dietary fiber obtained from extraction and compare this research with previous research to show readers how innovative techniques can improve dietary fiber properties.

-The impact of ultrasound extraction on increasing antioxidant capacity and an explanation of the relationship between structure and function should be included.

This will help highlight the optimization of extraction and the development of dietary fiber properties.

Conclusion: The conclusion should be written in a concise format. Summarize the key research findings, explain the meaning of the study results, suggest directions for future research, and identify the limitations of the study. This will provide readers with an overview of the research and highlight areas for future development and application.

Abbreviation: Should be added abbreviation section.

References: The citation format should be reviewed according to the journal's requirements. Furthermore, the most up-to-date citations should be selected.

**********

what does this mean?). If published, this will include your full peer review and any attached files.

If you choose “no”, your identity will remain anonymous but your review may still be made public.

**Do you want your identity to be public for this peer review?**  For information about this choice, including consent withdrawal, please see our Privacy Policy

Reviewer #1: No

**********

[NOTE: If reviewer comments were submitted as an attachment file, they will be attached to this email and accessible via the submission site. Please log into your account, locate the manuscript record, and check for the action link "View Attachments". If this link does not appear, there are no attachment files.]

To ensure your figures meet our technical requirements, please review our figure guidelines: https://journals.plos.org/plosone/s/figures

You may also use PLOS’s free figure tool, NAAS, to help you prepare publication quality figures: https://journals.plos.org/plosone/s/figures#loc-tools-for-figure-preparation.

NAAS will assess whether your figures meet our technical requirements by comparing each figure against our figure specifications.

# Author comment: Author response to Decision Letter 1

**DOI:** 10.1371/journal.pone.0357376.r002

17 Jul 2026

Review Comments to the Author:

Editor: The reply and manuscript’s text adjustments to the Editor´s comments were highlighted in yellow. We thank the editor for the thorough review of the manuscript.

• Novelty: Explicitly state what is new beyond applying known ultrasound and enzyme extraction to orange peel. Cite distinguishing factors in the final Introduction paragraph.

Reply: We added the following text to the final Introduction paragraph.

Moreover, evidence on the application of ultrasound as a pretreatment for SDF recovery from orange residues at low mass-to-volume ratios, such as 1:25, and under temperature-controlled sonication conditions remains limited. This mass-to-volume ratio offers a critical advantage by reducing solvent consumption and minimizing waste generation, thereby facilitating process scalability and industrial implementation. Beyond these operational advantages, the innovative contribution of this work lies in transcending the conventional physicochemical characterization by validating the biological functionality of the recovered SDF.

• Objective scope: Rewrite the objective to cover all treatments, including alkaline, ultrasound-alkaline, enzymatic, and UAEE, as well as all measured endpoints, including IDF, TDF, functional properties, structural properties, thermal properties, and bacterial growth.

Reply: We have modified the objective.

This study aims to evaluate the effects of different extraction methods (alkaline extraction, alkaline extraction with ultrasonic pretreatment, enzymatic extraction, and enzymatic extraction with ultrasonic pretreatment) on the extraction yield of dietary fiber fractions from OPB. The influence of these treatments on the techno-functional and structural properties of SFD was also assessed. Additionally, its potential as a carbon source for the growth of Faecalibaculum rodentium ABL288 was evaluated.

• Yield equation: Fix Equation (1) by providing a complete formula with denominator, define all terms, and specify whether the calculation is based on dry weight.

Reply: We checked Equation (1).

• Extraction conditions: Justify fixed ultrasound and enzymatic parameters, including 60% amplitude, pulse mode, temperature, and pH. State whether these were optimized, literature-based, or derived from preliminary trials.

Reply: Enzymatic extraction was performed following the methodology described by Kaur et al. [17], with some modifications. Ultrasound pretreatment conditions were derived from preliminary trials. This information was added to the text.

• Controls: Add untreated OPB, ultrasound-only, and enzyme-free buffer controls, or provide a clear justification for their omission.

Reply: We did not include untreated orange peel residues and enzyme-free buffer because the starting material underwent a preliminary treatment to remove low-molecular-weight sugars, organic acids, and non-fibrous soluble materials. Consequently, a buffer-only control would have provided additional data on the solubilization of the complex polysaccharide network that constitutes dietary fiber, since these compounds had already been eliminated. Moreover, the ultrasound-only control was omitted because, in this work, ultrasound was evaluated exclusively as a pretreatment to enhance mass transfer until the alkaline or enzymatic extraction processes. This approach aligns with the literature, which indicates that ultrasound typically enhances chemical or biological processes, thereby increasing fiber extraction.

• TDF reporting: Clarify whether total dietary fiber is analytically measured using AOAC 991.43 or calculated as SDF + IDF. Revise the column heading accordingly.

Reply: In Table 1, total dietary fiber was calculated as the sum of SDF and IDF. SDF purity was measured analytically using AOAC 991.43. For this analysis, only the SDF fraction was analyzed with the Sigma-Aldrich Total Dietary Fiber Assay Kit to determine SDF purity as the ratio of SDF to the SDF measured by AOAC 991.43. We have revised the column heading and modified the text to clarify this.

• SDF purity: Present the full SDF purity dataset, including the reported 85.51 to 92.15% range, in a table and include protein and ash corrections if applicable.

Reply: We added this information to the table.

• Replicates: Report biological and technical replicates explicitly for every assay, not only for the microbiology experiments.

Reply: The following information was added to the text.

All experiments were conducted in triplicate, except for WHC and OHC measurements, which were performed in duplicate.

• Statistics: Specify n-values, define whether data are presented as mean ± SD or mean ± SE, confirm ANOVA assumptions, and clarify letter groupings in all tables and figures.

Reply: We have clarified these parameters in the revised manuscript. All data are presented as mean ± standard deviation (SD). Independent experiments were performed in triplicate (n = 3), with the exception of water holding capacity (WHC) and oil holding capacity (OHC) measurements, which were conducted in duplicate (n = 2). The assumptions of normality, homogeneity of variances, and independence were verified prior to the analysis. Statistical significance was evaluated using one-way ANOVA followed by Tukey´s post hoc multiple comparisons test at a significance level of p < 0.05, in SPSS version 25. In all tables and figures, the presence of different lowercase letters indicates significant differences between treatment means.

• Units: Standardize all units, including rpm versus g, decimal and comma notation, and enzyme activity, according to SI units and journal style.

Reply: We have checked all units. However, we maintained the unit rpm (500 rpm) exclusively for agitation (stirring), as this refers to the rotational speed of the magnetic stirrer rather than a centrifugal separation force.

• Carbon-source inconsistency: Resolve conflicting glucose and SDF concentrations, 0.05% versus 0.5% w/v, in the MRS media description.

Reply: We acknowledge the reviewer's point and apologize for the inconsistency. We have harmonized the value describing the substrate concentration (0.5 g/L w/v) throughout the text.

• Positive control: Include a recognized prebiotic control, such as inulin, FOS, or GOS, for valid B. longum growth comparison, or substantially weaken the prebiotic claims.

Reply: We would like to notify you that, after a comprehensive review of our records regarding the culture setup and traceability, we have found that the mislabeling in the samples produced data shown in the original submission does not correspond to Bifidobacterium longum ABL182 cultures but rather to Faecalibaculum rodentium ABL288 cultures. F. rodentium is a mammal gastrointestinal symbiont belonging to the Erysipelothricaceae family and is closely related to Holdemanella biformis (doi: 10.1007/s10482-015-0583-3), a quite relevant human symbiont we have previously characterized (doi: 10.1096/fj.202100126R). We have corroborated via molecular markers (16S rRNA gene sequencing) that the culture was pure, and we have retained the correctly identified data and added new replicates that are consistent with previous data. Moreover, we added novel information on the culture conditions of a mammal and human symbiont, recognized as a paradigm of glycan utilization versatility, Bacteroides thetaiotaomicron (doi: 10.1186/1471-2164-14-873). The response of B. thetaiotaomicron to OPB-derived fiber has been distinct from that of F. rodentium, confirming its high capacity for glycan utilization and showing better, though not significant, growth rates under glucose conditions. Consequently, we have modified the text to reflect this new configuration of microbiology assays, while toning down the meaning of our findings to avoid overclaiming (lines 206-224, 512-252, 539-555).

• Overclaiming: Revise the abstract, discussion, and conclusion because SDF-UAEE biomass of approximately 6.1% relative to glucose indicates limited substrate utilization rather than strong prebiotic potential.

Reply: We have toned down our overclaiming and aligned the conclusions with the scope of the results.

• Metabolic evidence: Supplement OD₆₀₀ data with SCFA production, pH reduction, or carbohydrate-consumption data. At minimum, acknowledge the lack of metabolic evidence as a key limitation.

Reply: Such limitation was explicitly stated in the conclusion section.

• TGA inconsistency: Correct whether thermogravimetric analysis was performed on SDF or IDF, and revise the section and figure caption consistently.

Reply: Thermogravimetric analysis was performed on SDF. This information was clarified in the text.

• XRD quantification: Calculate and report a crystallinity index or quantitative peak analysis instead of relying solely on visual interpretation.

Reply: We added the crystallinity index to the text using the following methodology:

The degree of crystallinity was evaluated using the crystallinity index, calculated according to equation (4) described by de Miranda et al. [25].

Crystallinity index (%)= (I_002- I_am)/I_002 *100

where I002 is the diffraction peak intensity of the crystalline material near 2θ = 20° due to the nature of the soluble fiber, and Iam is the diffraction peak intensity of the amorphous material near 2θ = 15°. The values obtained from the crystallinity index are relative measures of molecular ordering in the polymer matrix.

Moreover, the following discussion was added in the results and discussion section:

As shown in Fig. 4, the SDF obtained by ALE has the highest crystallinity index (36%), whereas the SDF obtained by UAEE has the lowest (5%). The decrease in the crystallinity index among the treatments was mainly attributed to the broadening of the crystalline peak around 20.4° and the absence of cellulose peaks at 20.0°–22.0°, confirming the impact of ultrasound on the crystalline lattice. These results align with those reported by Yang et al. [68], who found that ultrasonic waves weakened intermolecular interactions and reduced the crystallinity index from 23.28% to 17.65% after ultrasound treatment of SDF obtained from grapefruit peels. A similar effect was reported by Zhou et al. [30]. They found that the crystallinity index of SDF obtained using deep eutectic solvents combined with ultrasonic pretreatment decreased from 31.48% to 29.73% after ultrasound treatment, possibly due to disruption of cellulose chains by ultrasonic cavitation.

• FTIR interpretation: Provide a table of major bands, assignments, and peak shifts or intensity changes for each treatment instead of relying on a purely descriptive narrative.

Reply: We added the values corresponding to the peak wavenumbers of the functional groups (cm-1) for the different treatments in Table 3 below:

As can be observed in Table 3, all samples exhibit the characteristic adsorption bands of SDF components such as cellulose, hemicellulose, and pectin. Specifically, the presence of peaks near 2926 - 2929 cm-1 (C– H stretching in methylene groups) and 1595 - 1626 cm-1 (carbonyl groups) confirms that the core chemical identity of the orange peel remains intact regardless of the extraction method used. Moreover, the shift in the hydroxyl group (-OH) from 3256 cm-1 (ALE) to 3275 cm-1 (UAEE) indicates a decrease in intermolecular hydrogen bonding. This suggests that the combined ultrasound pretreatment and enzymatic extraction effectively disrupted the plant matrix, making the fiber structure more porous compared with the conventional chemical extraction methods.

Table 3. FTIR spectral assignments and characteristic peak wavenumbers (cm⁻¹) of SDF obtained from OPB using different treatments.

Functional group Characteristic molecular vibration ALE UAEAL EE UAEE

Hydroxyl (−OH) O–H stretching of hydroxyl groups associated with hemicellulose and pectin 3256 3275 3275 3275

Alkanes (C−H) C-H stretching of methyl and methylene groups in polysaccharides 2929 2926 2928 2929

Carbonyl (C=O) Stretching vibration of esterified carbonyl groups 1595 1595 1619 1626

Alkanes/ Acids (C−H / C−O) Symmetric bending and stretching vibration of polysaccharides 1404 1406 1389 1395

Backbones (C−O−C / C−O) Stretching vibration of saccharide units or piranose rings 1004 1013 1055 1061

• Figure 4 caption: Correct SEM labels so that A to D correspond to ALE, UAEAL, EE, and UAEE, respectively, and fix all omissions and misalignments in the caption.

Reply: We checked the Figure caption.

• Industrial claims: Reframe food-fortification and bakery shelf-life statements as future hypotheses because no food-system experiments were conducted.

Reply: We have reframed the section as follows:

Given these functional properties, the SDF could have industrial applications. For instance, the fibers obtained through ALE and UAEAL are hypothesized to have applications in bakery and pastry products by promoting moisture retention and influencing freshness and shelf-life characteristics. This hypothesis is supported by studies showing that fiber integration can improve texture and moisture retention in baked goods [26, 46]. Furthermore, the SDF fractions could potentially serve as fat replacers in dairy, meat, and emulsified food systems, although this remains to be experimentally validated in future studies. These properties suggest that the recovered fibers are promising candidates for functional food development, though further research is required to evaluate their performance in specific food systems.

• Language and formatting: Submit the manuscript for professional English editing and correct grammar errors, inconsistent capitalization, and typographical errors, including “he application,” “micrography,” and “amorphous status.”.

Reply: We have checked the manuscript using Grammarly Pro and with a native English-speaking colleague. We hope the manuscript meets the requirements.

Reviewer #1: The reply and manuscript’s text adjustments to Reviewer #1's comments were highlighted in green. We thank the Reviewer for the thorough review of the manuscript.

Introduction: The introduction should be reviewed to cover the following key points and should relate them to current updated research. should be given regarding:

• The problem and its significance should be introduced in a way that reflects the current situation, helping readers understand the motivation behind the research and highlighting the importance of utilizing value-added materials.

Reply: Thank you for your comment. We have added the following text to improve the introduction.

Orange is one of the most important crops worldwide, with an estimated annual production of 47 million tons in 2024 [1]. Globally, the management of citrus residues is a critical environmental challenge, as millions of tons of organic waste are generated each year, releasing significant amounts of greenhouse gases, including carbon dioxide and methane, during decomposition [2]. These by-products, which include peel, albedo, and endocarps, account for up to 55% of the fruit's weight. In Colombia, where production reached 756 thousand tons in 2023, the common practice of discarding these residues in landfills represents a substantial loss of biomass rich in carotenoids, flavonoids, pectin, and dietary fiber (DF) [3]. Consequently, in accordance with the principles of the circular economy, it is imperative to develop valorization strategies that mitigate environmental impacts while recovering high-value functional ingredients [4].

• Detailed technical information on extraction methods should be included, along with introductory information on the research methods, to help readers understand the relevant innovations and technologies.

Reply: Thank you for the comment. We have added the following text to improve the introduction.

Traditional chemical extraction methods (acidic or alkaline) are common due to low cost but often require high temperatures and pose environmental challenges [12]. In contrast, ultrasound-assisted extraction (UAE) is a sustainable “green” technology that operates at lower temperatures with reduced solvent requirements [13]. The core mechanism is acoustic cavitation: sound waves (typically> 20 kHz) c

Attachment Submitted filename: Response to Reviewers.docx

# Editor report: Decision Letter 1

**DOI:** 10.1371/journal.pone.0357376.r003

**Authors:** Karthikeyan Venkatachalam

31 Jul 2026

Dear Dr. Osorio-Tobón,

Thank you for submitting your manuscript to PLOS ONE. After careful consideration, we feel that it has merit but does not fully meet PLOS ONE’s publication criteria as it currently stands. Therefore, we invite you to submit a revised version of the manuscript that addresses the points raised during the review process.

Please submit your revised manuscript by Sep 14 2026 11:59PM. If you will need more time than this to complete your revisions, please reply to this message or contact the journal office at plosone@plos.org. When you're ready to submit your revision, log on to https://www.editorialmanager.com/pone/ and select the 'Submissions Needing Revision' folder to locate your manuscript file.

- A letter that responds to each point raised by the academic editor and reviewer(s). You should upload this letter as a separate file labeled 'Response to Reviewers'.
- A marked-up copy of your manuscript that highlights changes made to the original version. You should upload this as a separate file labeled 'Revised Manuscript with Track Changes'.
- An unmarked version of your revised paper without tracked changes. You should upload this as a separate file labeled 'Manuscript'.

If you would like to make changes to your financial disclosure, please include your updated statement in your cover letter. Guidelines for resubmitting your figure files are available below the reviewer comments at the end of this letter.

If applicable, we recommend that you deposit your laboratory protocols in protocols.io to enhance the reproducibility of your results. Protocols.io assigns your protocol its own identifier (DOI) so that it can be cited independently in the future. For instructions see: https://journals.plos.org/plosone/s/submission-guidelines#loc-laboratory-protocols. Additionally, PLOS ONE offers an option for publishing peer-reviewed Lab Protocol articles, which describe protocols hosted on protocols.io. Read more information on sharing protocols at https://plos.org/protocols?utm_medium=editorial-email&utm_source=authorletters&utm_campaign=protocols.

As the corresponding author, your ORCID iD is verified in the submission system and will appear in the published article. PLOS supports the use of ORCID, and we encourage all coauthors to register for an ORCID iD and use it as well. Please encourage your coauthors to verify their ORCID iD within the submission system before final acceptance, as unverified ORCID iDs will not appear in the published article. *Only*  the individual author can complete the verification step; PLOS staff *cannot*  verify ORCID iDs on behalf of authors.

We look forward to receiving your revised manuscript.

Kind regards,

Karthikeyan Venkatachalam, Ph.D.

Academic Editor

PLOS One

Journal Requirements:

1. If the reviewer comments include a recommendation to cite specific previously published works, please review and evaluate these publications to determine whether they are relevant and should be cited. There is no requirement to cite these works unless the editor has indicated otherwise.

2. Please review your reference list to ensure that it is complete and correct. If you have cited papers that have been retracted, please include the rationale for doing so in the manuscript text, or remove these references and replace them with relevant current references. Any changes to the reference list should be mentioned in the rebuttal letter that accompanies your revised manuscript. If you need to cite a retracted article, indicate the article’s retracted status in the References list and also include a citation and full reference for the retraction notice.

Additional Editor Comments:

• The conclusions contain an important inconsistency. The manuscript states that enzymatic extraction represents the best extraction method due to its higher yield and purity, while later concluding that ultrasound-pretreated enzymatic extraction demonstrated the highest extraction yield and the most promising structural and functional properties. Please reconcile these statements and clearly identify the most suitable extraction method based on the overall results.

• Several statements regarding industrial applicability appear stronger than the experimental evidence supports. Claims such as “technical feasibility for industrial-scale application” and “strong potential for industrial application” should be moderated unless supported by pilot-scale, techno-economic, or scale-up data.

• The discussion of the prebiotic potential should be presented more cautiously. The current findings demonstrate that the recovered fiber can support the growth of Bifidobacterium longum under the tested conditions, but broader health, microbiome, or functional claims require additional metabolic, genomic, or in vivo evidence.

• Please improve several sentences in the Conclusions section that are difficult to follow grammatically. For example, expressions such as “transferring a greater amount of stabilizing capacity fibers” and “demonstrated the highest extraction yield and the most promising in structural and functional properties” should be revised for clarity and readability.

• The statement that EE provides the highest yield should be carefully checked against the reported data showing that UAEE achieved the highest SDF recovery (26.04 ± 0.81%). The interpretation of yield versus purity should be clarified throughout the manuscript.

• The manuscript frequently refers to enhanced antioxidant potential based on higher phenolic and flavonoid contents. Please ensure that all antioxidant-related statements are restricted to the measured parameters and do not imply biological activity beyond the available evidence.

• Please consider adding a brief statement acknowledging that further studies are required to evaluate process scalability, economic feasibility, and performance in real food systems before industrial implementation.

• A final thorough English language revision is recommended to improve sentence structure, reduce awkward phrasing, and enhance overall readability. While the scientific content is generally sound, linguistic polishing would significantly improve the manuscript.

[Note: HTML markup is below. Please do not edit.]

[NOTE: If reviewer comments were submitted as an attachment file, they will be attached to this email and accessible via the submission site. Please log into your account, locate the manuscript record, and check for the action link "View Attachments". If this link does not appear, there are no attachment files.]

To ensure your figures meet our technical requirements, please review our figure guidelines: https://journals.plos.org/plosone/s/figures

You may also use PLOS’s free figure tool, NAAS, to help you prepare publication quality figures: https://journals.plos.org/plosone/s/figures#loc-tools-for-figure-preparation.

NAAS will assess whether your figures meet our technical requirements by comparing each figure against our figure specifications.

# Author comment: Author response to Decision Letter 2

**DOI:** 10.1371/journal.pone.0357376.r004

5 Aug 2026

Review Comments to the Author:

Editor: The reply and manuscript’s text adjustments to the Editor´s comments were highlighted in yellow. We thank the editor for the thorough review of the manuscript.

• The conclusions contain an important inconsistency. The manuscript states that enzymatic extraction represents the best extraction method due to its higher yield and purity, while later concluding that ultrasound-pretreated enzymatic extraction demonstrated the highest extraction yield and the most promising structural and functional properties. Please reconcile these statements and clearly identify the most suitable extraction method based on the overall results.

Reply: We agree with the Editor´s comments regarding the terminology. In the revised manuscript, we have clarified that the enzymatic methods (as a category) are superior to conventional alkaline extraction in terms of yield and purity; the ultrasound-pretreated enzymatic method is identified as the most suitable method overall. This is because UAEE achieved the significantly highest yield (26.04%), while maintaining a high purity level (91.09%) that is statistically comparable to EE treatment. Furthermore, UAEE demonstrated superior performance in TPC and TFP, and thermal stability, as now stated in the updated Conclusions section.

• Several statements regarding industrial applicability appear stronger than the experimental evidence supports. Claims such as “technical feasibility for industrial-scale application” and “strong potential for industrial application” should be moderated unless supported by pilot-scale, techno-economic, or scale-up data.

Reply: Thank you for your comment. We have moderated our claims to ensure they are strictly supported by our experimental evidence.

• The discussion of the prebiotic potential should be presented more cautiously. The current findings demonstrate that the recovered fiber can support the growth of Bifidobacterium longum under the tested conditions, but broader health, microbiome, or functional claims require additional metabolic, genomic, or in vivo evidence.

Reply: Thank you for your comment. We have moderated the discussion and conclusions to reflect the preliminary nature of these findings.

• Please improve several sentences in the Conclusions section that are difficult to follow grammatically. For example, expressions such as “transferring a greater amount of stabilizing capacity fibers” and “demonstrated the highest extraction yield and the most promising in structural and functional properties” should be revised for clarity and readability.

Reply: Thank you for your comment. We have rewritten the conclusions section.

• The statement that EE provides the highest yield should be carefully checked against the reported data showing that UAEE achieved the highest SDF recovery (26.04 ± 0.81%). The interpretation of yield versus purity should be clarified throughout the manuscript.

Reply: We thank the editor for pointing out this inconsistency. We have carefully checked our data and clarified the distinction between the general enzymatic approach and the optimized UAEE protocol.

• The manuscript frequently refers to enhanced antioxidant potential based on higher phenolic and flavonoid contents. Please ensure that all antioxidant-related statements are restricted to the measured parameters and do not imply biological activity beyond the available evidence.

Reply: We thank the editor for this clarification. We have updated all antioxidant-related statements to ensure they are strictly restricted to the measured in vitro parameters.

• Please consider adding a brief statement acknowledging that further studies are required to evaluate process scalability, economic feasibility, and performance in real food systems before industrial implementation.

Reply: Thank you for the comment. We have added the required statement at the end of the Conclusions section.

• A final thorough English language revision is recommended to improve sentence structure, reduce awkward phrasing, and enhance overall readability. While the scientific content is generally sound, linguistic polishing would significantly improve the manuscript.

Reply: We have completed the final language revision. We hope this time we meet the requirements.

Attachment Submitted filename: Response_to_Reviewers_auresp_2.docx

# Editor report: Decision Letter 2

**DOI:** 10.1371/journal.pone.0357376.r005

**Authors:** Karthikeyan Venkatachalam

17 Aug 2026

Valorization of orange peel by-products via ultrasound-pretreated enzymatic extraction of soluble dietary fiber: structural and functional characterization

PONE-D-26-07738R2

Dear Dr. Osorio-Tobón,

We’re pleased to inform you that your manuscript has been judged scientifically suitable for publication and will be formally accepted for publication once it meets all outstanding technical requirements.

Within one week, you’ll receive an e-mail detailing the required amendments. When these have been addressed, you’ll receive a formal acceptance letter and your manuscript will be scheduled for publication.

An invoice will be generated when your article is formally accepted. Please note, if your institution has a publishing partnership with PLOS and your article meets the relevant criteria, all or part of your publication costs will be covered. Please make sure your user information is up-to-date by logging into Editorial Manager at Editorial Manager® and clicking the ‘Update My Information' link at the top of the page. For questions related to billing, please contact billing support.

If your institution or institutions have a press office, please notify them about your upcoming paper to help maximize its impact. If they’ll be preparing press materials, please inform our press team as soon as possible -- no later than 48 hours after receiving the formal acceptance. Your manuscript will remain under strict press embargo until 2 pm Eastern Time on the date of publication. For more information, please contact onepress@plos.org.

Kind regards,

Karthikeyan Venkatachalam, Ph.D.

Academic Editor

PLOS One

Additional Editor Comments (optional):

Authors have addressed all the given comments.

This paper can be accepted for publication.

Reviewers' comments:

# Editor report: Acceptance letter

**DOI:** 10.1371/journal.pone.0357376.r006

**Authors:** Karthikeyan Venkatachalam

PONE-D-26-07738R2

PLOS One

Dear Dr. Osorio-Tobón,

I'm pleased to inform you that your manuscript has been deemed suitable for publication in PLOS One. Congratulations! Your manuscript is now being handed over to our production team.

At this stage, our production department will prepare your paper for publication. This includes ensuring the following:

* All references, tables, and figures are properly cited

* All relevant supporting information is included in the manuscript submission,

* There are no issues that prevent the paper from being properly typeset

You will receive further instructions from the production team, including instructions on how to review your proof when it is ready. Please keep in mind that we are working through a large volume of accepted articles, so please give us a few days to review your paper and let you know the next and final steps.

Lastly, if your institution or institutions have a press office, please let them know about your upcoming paper now to help maximize its impact. If they'll be preparing press materials, please inform our press team within the next 48 hours. Your manuscript will remain under strict press embargo until 2 pm Eastern Time on the date of publication. For more information, please contact onepress@plos.org.

You will receive an invoice from PLOS for your publication fee after your manuscript has reached the completed accept phase. If you receive an email requesting payment before acceptance or for any other service, this may be a phishing scheme. Learn how to identify phishing emails and protect your accounts at https://explore.plos.org/phishing.

If we can help with anything else, please email us at customercare@plos.org.

Thank you for submitting your work to PLOS One and supporting open access.

Kind regards,

PLOS One Editorial Office Staff

on behalf of

Dr. Karthikeyan Venkatachalam

Academic Editor

PLOS One
